# Transcriptome analysis of *Edwardsiella piscicida* during intracellular infection reveals excludons are involved with the activation of a mitochondrion-like energy generation program

**DOI:** 10.1128/mbio.03526-23

**Published:** 2024-02-13

**Authors:** Andre Lanza, Satoshi Kimura, Ikuo Hirono, Kazutoshi Yoshitake, Shigeharu Kinoshita, Shuichi Asakawa

**Affiliations:** 1 Department of Aquatic Bioscience, Graduate School of Agricultural and Life Sciences, The University of Tokyo, Tokyo, Japan; 2 Department of Biomaterial Sciences, Graduate School of Agricultural and Life Sciences, The University of Tokyo, Tokyo, Japan; 3 Department of Marine Biosciences, Graduate School of Marine Science and Technology, Tokyo University of Marine Science and Technology, Tokyo, Japan; University of Nebraska Medical Center, Omaha, Nebraska, USA

**Keywords:** magnetic nanoparticles, transcriptomics, bioenergetics, excludon, antisense RNA, mitochondria, intracellular bacteria, tricarboxylic acid cycle, proteobacteria

## Abstract

Phylogenetic evidence suggests a shared ancestry between mitochondria and modern Proteobacteria, a phylum including several genera of intracellular pathogens. Studying these diverse pathogens, particularly during intracellular infection of their hosts, can reveal characteristics potentially representative of the mitochondrial-Proteobacterial ancestor by identifying traits shared with mitochondria. While transcriptomic approaches can provide global insights into intracellular acclimatization by pathogens, they are often limited by excess host RNAs in extracts. Here, we developed a method employing magnetic nanoparticles to enrich RNA from an intracellular Gammaproteobacterium, *Edwardsiella piscicida*, within zebrafish, *Danio rerio*, fin fibroblasts, enabling comprehensive exploration of the bacterial transcriptome. Our findings revealed that the intracellular *E. piscicida* transcriptome reflects a mitochondrion-like energy generation program characterized by the suppression of glycolysis and sugar transport, coupled with upregulation of the tricarboxylic acid (TCA) cycle and alternative import of simple organic acids that directly flux into TCA cycle intermediates or electron transport chain donors. Additionally, genes predicted to be members of excludons, loci of gene pairs antagonistically co-regulated by overlapping antisense transcription, are significantly enriched in the set of all genes with perturbed sense and antisense transcription, suggesting a general but important involvement of excludons with intracellular acclimatization. Notably, genes involved with the activation of the mitochondrion-like energy generation program, specifically with metabolite import and glycolysis, are also members of predicted excludons. Other intracellular Proteobacterial pathogens appear to employ a similar mitochondrion-like energy generation program, suggesting a potentially conserved mechanism for optimized energy acquisition from hosts centered around the TCA cycle.

**IMPORTANCE:**

Phylogenetic evidence suggests that mitochondria and Proteobacteria, a phylum encompassing various intracellular pathogens, share a common ancestral lineage. In this study, we developed a novel method employing magnetic nanoparticles to explore the transcriptome of an aquatic Gammaproteobacterium, *Edwardsiella piscicida*, during intracellular infection of host cells. We show that the strategy *E. piscicida* uses to generate energy strikingly mirrors the function of mitochondria—energy generators devoid of glycolytic processes. Notably, several implicated genes are members of excludons—gene pairs antagonistically co-regulated by overlapping antisense transcription. Other intracellular Proteobacterial pathogens appear to adopt a similar mitochondrion-like energy generation program, indicating a possibly conserved strategy for optimized energy acquisition from hosts centered around the tricarboxylic acid cycle.

## INTRODUCTION

The intracellular internalization of prokaryotes by eukaryotes whether through predation or by infection has been a major theme influencing the evolution of both groups since their first divergence. In one unique case, the internalization of a Proteobacterium has led to the origin of mitochondria (1–3), an “eternal” symbiosis and catalyst for the evolution of the complex multicellular life observed today. In other cases, internalization has led to the extinction of a plethora of both prokaryotic and eukaryotic species that will remain unknown to us. For the prokaryote, the intracellular environment is hostile as the host actively attempts to destroy it unless some mutual benefit is leveraged (4). Consequently, its survival depends on its ability to escape whether by enduring until its extrusion or by killing the eukaryote. This ability is endowed to the prokaryote by the timely and concerted expression of genes that counteract the host’s assault. The ebb and flow of the conflict between the two groups over the eons has resulted in the evolution of highly specialized intracellular bacterial pathogens that cause significant diseases affecting human, agricultural, and wildlife health in modern day (5, 6). The study of these diverse pathogens particularly during intracellular infection of their hosts will continue to provide valuable insight into the co-evolution of both groups and can guide the development of novel disease therapies by targeting common intracellular survival strategies.

*Edwardsiella piscicida* is a Gram-negative, aquatic Gammaproteobacterium that infects diverse marine and freshwater animals and is most notorious for its impact on fish farming economics globally (7). Central to *E. piscicida* pathogenesis is the ability to invade phagocytic and non-phagocytic cells and to replicate within specialized vacuoles mediated by type III and type VI secretion systems (T3SS/T6SS) (8–10). Although the *E. piscicida* secretion systems and their effector proteins are under intense study, there has yet to be a report on its transcriptional landscape during intracellular infection, which would provide overarching insights into its mechanisms of immune evasion, metabolic acclimatization to the intracellular niche, and nutrient acquisition from the host. Previous attempts to decipher the intracellular-acclimatized transcriptome have involved culturing *E. piscicida* in Dulbecco’s modified Eagle’s medium to mimic intracellular conditions (11). Despite these efforts, the knowledge gap regarding a fully representative intracellular-acclimatized transcriptome remains and can largely be attributed to the difficulty of isolating sufficient bacterial RNA from infected cells for sequencing to a level adequate for comprehensive gene expression analysis (12).

There is mounting evidence that pervasive antisense transcription (PAT) of coding sequences is deeply involved with the regulation of gene expression (13). It has been proposed that PAT arising from the overlapping transcription of (transcriptionally) convergent and divergent gene pairs can lead to the antagonistic co-regulation of the pairs in a system termed as an excludon (14). In some cases, these excludons can even exist as bistable switches controlling for the respective “on” and “off” states of the respective genes (15). Genome size reduction is widespread in intracellular pathogens (16), and gene regulation through PAT and excludons may become increasingly vital for intracellular adaptation as coding and intergenic spaces are gradually reduced. Here, we report the development of a novel method employing magnetic nanoparticles (MNPs) to rapidly separate *E. piscicida* from infected zebrafish, *Danio rerio*, fin fibroblasts, enabling enrichment of bacterial RNA and thus facilitating exploration of the bacterial transcriptome. We could comprehensively interrogate not only sense transcripts but also PAT and found strong evidence for a general but important role of excludons in *E. piscicida*’s transcriptional activation of a mitochondrion-like energy generation program.

## RESULTS

### Rapid magnetic separation of *E. piscicida* from host lysates

**Fig 1:**
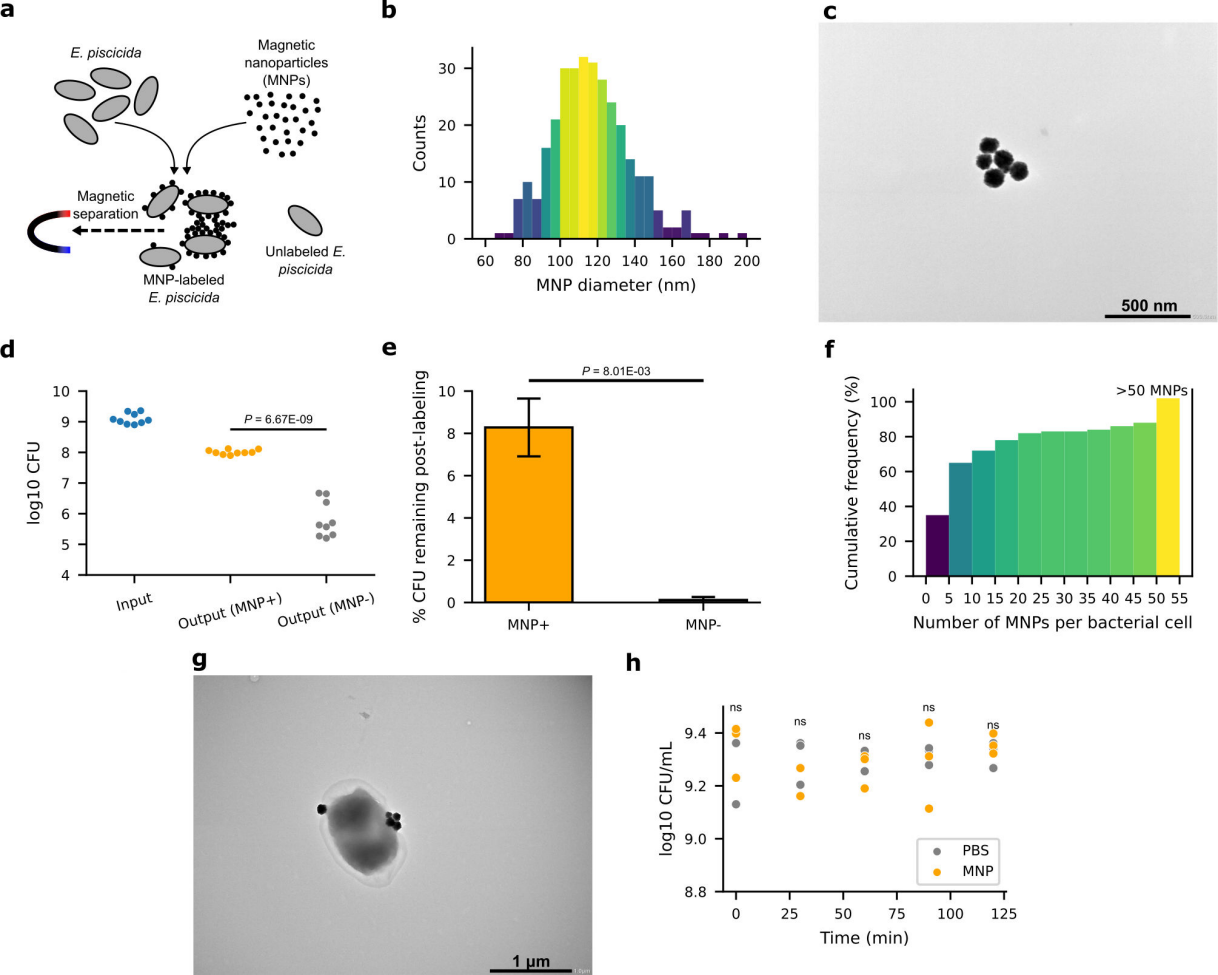
MNPs bind to the surface of *E. piscicida* without affecting its viability. (**a)** Schematic showing the labeling of *E. piscicida* with MNPs and their separation from unlabeled bacteria by magnetic capture. (**b and c)** Size distribution (**b**) and TEM micrograph (**c**) of MNPs in the supernatant of a diluted, sonicated, and centrifuged commercial stock solution. *n =* 312 MNPs. (**d)** Log10 total colony-forming units (CFU) before (input, blue) and remaining after (output) magnetic capture of *E. piscicida* incubated with MNPs (MNP+, orange) or phosphate-buffered saline (PBS) (MNP−, gray). (**e)** Percentage of initial input CFU remaining after magnetic capture of MNP-labeled (MNP+) or mock-labeled (MNP−) *E. piscicida* from panel **d**. **(f)** Cumulative frequency of the number of MNPs labeled to the surface of bacterial cells. Labeled MNPs exceeding 50 are grouped into one bin. *n =* 102 bacteria. (**g)** Representative TEM micrograph of an *E. piscicida* cell with five MNPs labeled to its surface. (**h)** Log10 viable CFU sampled over 2 h of incubation of *E. piscicida* with MNPs (orange) or PBS (gray). Data points in panel **d** are from three independent experiments, each performed with three biological replicates. Shown in panel **e** are the mean ± s.d. of the means from three independent experiments. Data points in panel **h** are from three biological replicates at each time point. Scale bars: (**c)** 500 nm; (**g)** 1 µm. Data were analyzed using two-tailed unpaired Student’s *t*-test (**d, e, and h**). *P* < 0.05 are displayed and otherwise marked as ns.

Our approach for bacterial RNA enrichment from infected cells was by a differential lysis method with the aim of limiting the potential artifacts of sample manipulation on bacterial gene expression. Specifically, we sought to minimize the time between host lysis and RNA extraction from the bacteria while preserving the integrity of the bacteria-containing vacuole. To this end, we labeled *E. piscicida* EtPo1 with MNPs (Fig. 1a) prior to infecting zebrafish fin fibroblasts, which would allow for their rapid separation from host lysates by an external magnetic field. Additionally, we employed digitonin for host lysis, which selectively lyses cholesterol-rich plasma membranes while maintaining the integrity of intracellular membranes with low cholesterol content (17, 18).

The attachment of nanoparticles to bacteria has been attributed to their surface charges and can be explained by the Derjaguin-Landau-Vervey-Overbeek (DLVO) theory, which models particle aggregation in a liquid medium (19, 20). We performed labeling of *E. piscicida* with MNPs as previously described for *Streptococcus pyogenes* (21) with some modifications (see Materials and Methods). A short 30-minute incubation of size-fractionated MNPs having a mean diameter of 116.7 nm (Fig. 1b and c) and *E. piscicida* at a ratio of approximately five MNPs to one bacterium allowed us to recover about 8% (Fig. 1d and e) of the initial bacteria input on average after magnetic capture and washing on a magnetic rack. This ratio was chosen to minimize particle crowding on the bacterial surface, which might inhibit bacteria-host membrane interactions required during invasion and intracellular infection. Transmission electron microscopy (TEM) of labeled bacteria showed that about 65% of counted bacteria was labeled with between 1 and 10 MNPs although occasionally large aggregates attached to bacterial surfaces could be observed (Fig. 1f and g; Fig. S1). As there have been reports of nanoparticles exhibiting antibiotic activity against various bacteria (22), we extended the incubation and assessed bacterial viability over a 2-h period. There was no obvious effect on the viability of *E. piscicida* (Fig. 1h) likely owing to the MNPs’ relatively, chemically inert polystyrene coating. MNP labeling also showed no adverse effects on the quality of extracted RNA (Fig. S2).

**Fig 2:**
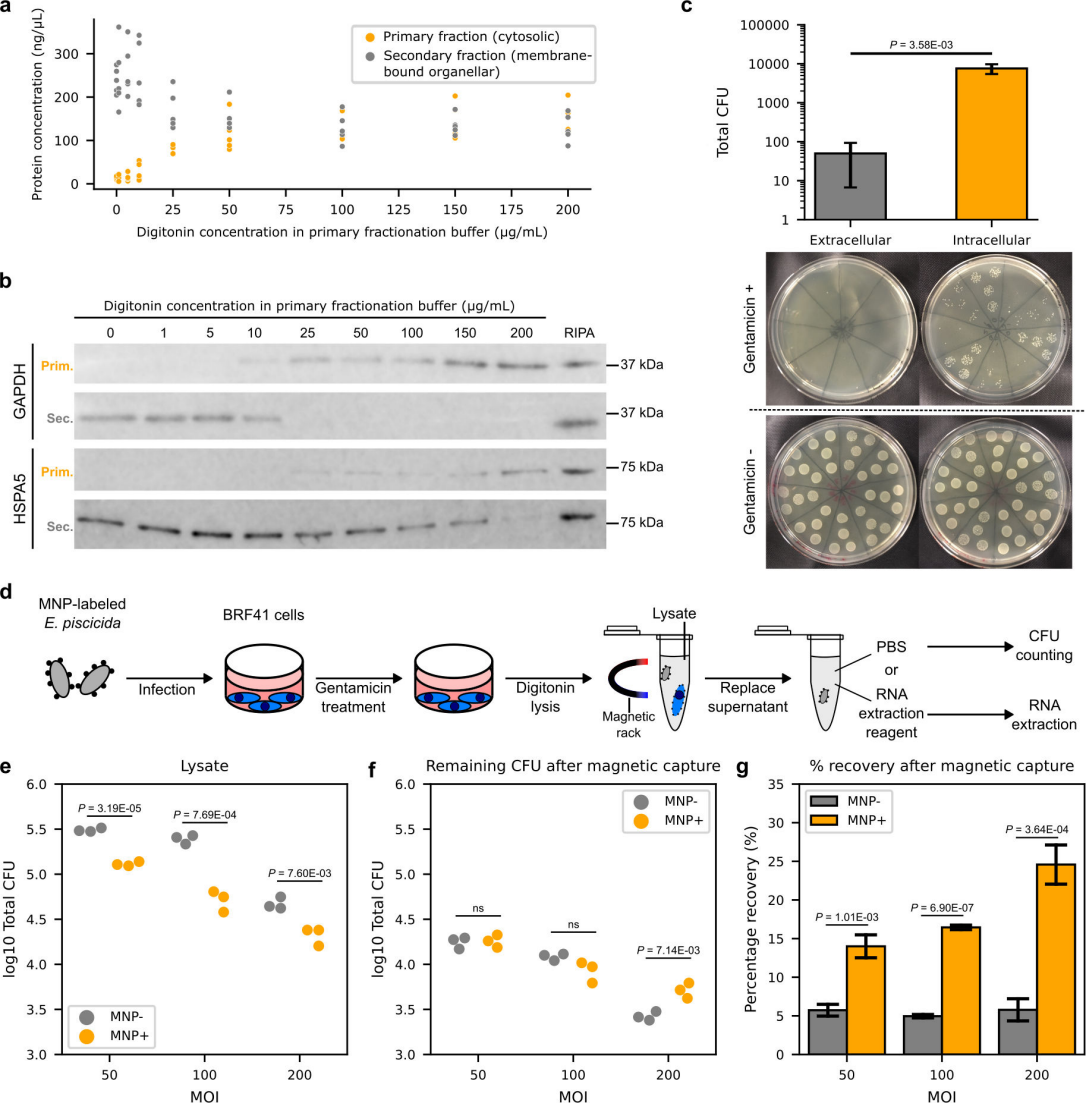
MNP-labeled *E. piscicida* can be magnetically separated from zebrafish fibroblast lysates. (**a)** Protein concentrations in the subcellular fractions of sequentially fractionated zebrafish fibroblasts primarily lysed with varying concentrations of digitonin. (**b)** Western blot of cytosolic glyceraldehyde-3-phosphate dehydrogenase, GAPDH, and the ER lumen protein, HSPA5/BiP, in the primary (Prim.) and secondary (Sec.) subcellular fractions of zebrafish fibroblasts primarily lysed with varying concentrations of digitonin. Whole-cell lysates in RIPA buffer were loaded as a control. (**c)** Total colony-forming unit (CFU) in the cell culture medium (extracellular) and lysates (intracellular) of zebrafish fibroblasts infected with *E. piscicida* at a multiplicity of infection (MOI) of 50 and treated with gentamicin for 3 h. The extracellular and intracellular CFU in non-treated cells (Gentamicin−) were innumerable at the same dilutions used to count CFUs from treated cells (Gentamicin+). (**d)** Schematic showing the procedure of infecting zebrafish fibroblasts with MNP-labeled *E. piscicida* and their subsequent separation from host lysates by a magnetic rack for CFU counting or RNA extraction. (**e)** Log10 total CFU in the lysates of zebrafish fibroblasts infected with MNP-labeled (MNP+) or unlabeled (MNP−) *E. piscicida* at MOIs of 50, 100, or 200. (**f)** Remaining CFU after magnetic capture of lysates in panel **e**. (g) Percentage of recovery of CFU after magnetic capture of lysates from panels **e** and **f**. Data points in panel **a** are from five independent experiments. Data points in panels **e** and **f** are from three biological replicates. Shown in panels **c** and **g** are the mean ± s.d. of three biological replicates. Data were analyzed using two-tailed unpaired Student’s *t*-test (**c, e–g**). *P* < 0.05 are displayed and otherwise marked as ns.

Following a previous study employing digitonin in the sequential, subcellular fractionation of mammalian cells (23), we found that digitonin at a concentration of 25 µg/mL in the primary fractionation buffer also effectively lysed the plasma membrane of zebrafish fibroblasts while maintaining the integrity of the organellar membranes as shown in western blot analysis of cytosolic and endoplasmic reticulum (ER) lumen proteins in different lysate fractions (Fig. 2a and b). We next established an intracellular infection model of *E. piscicida* in zebrafish fin fibroblasts, BRF41 cells, coinciding with the early phase of intracellular replication after entry into host cells as reported in ZF4 cells, a similar zebrafish fin fibroblast cell line (24). We accomplished this using a gentamicin invasion assay (25) and confirmed the elimination of virtually all extracellular bacteria in the cell culture medium (Fig. 2c). Finally, combining all procedures into one, we tested if bacteria labeled with MNPs were able to invade host cells and if they could be separated from the lysates by magnetic capture (Fig. 2d). Bacteria were quantified before and after magnetic separation from the lysate, and our findings confirmed that, first, MNP-labeled bacteria were indeed able to invade host cells albeit with lower efficiency compared to unlabeled bacteria (Fig. 2e), and second, a significantly higher percentage of labeled bacteria could be magnetically separated from the lysate compared to unlabeled bacteria (Fig. 2f and g). MNP labeling might negatively impact effector secretion as indirectly inferred from MNP-labeled bacteria exhibiting a lower invasion capability, which is dependent on effector secretion. Nonetheless, labeled bacteria were able to establish infection to a high degree suggesting near full functionality of the secretion systems and the feedback systems that feed into them.

### Bacterial RNA enrichment improves read mapping to the bacterial genome

**Fig 3:**
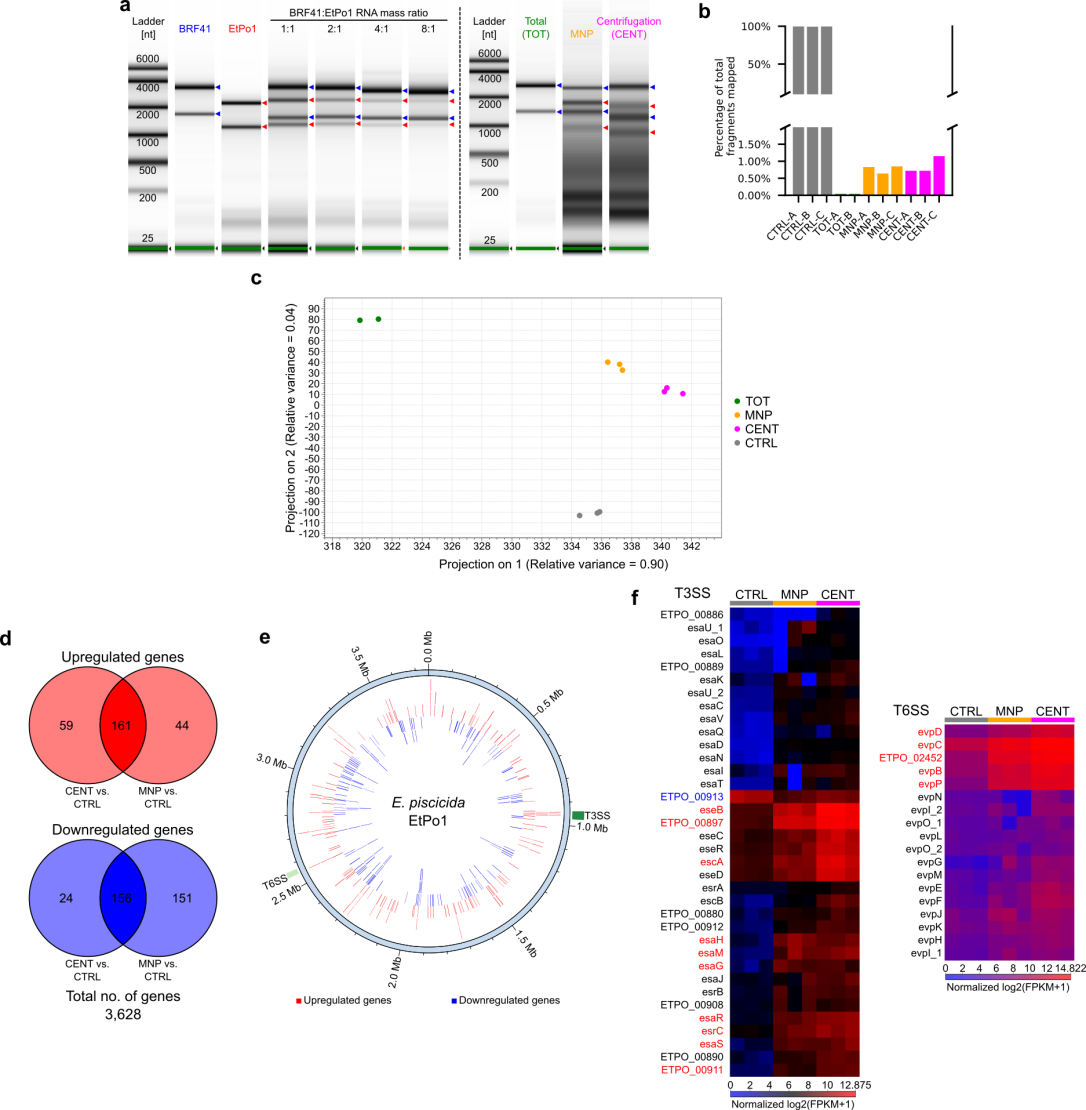
Enrichment of *E. piscicida* RNA from infected zebrafish fibroblasts allows comprehensive analysis of gene expression. (**a)** Electropherogram of pure *E. piscicida* (EtPo1) and zebrafish fibroblast (BRF41) RNA and mock mixed RNA at host:bacteria mass ratios of 1:1, 2:1, 4:1, and 8:1 (left panel). Representative electropherogram of RNA extracted from infected BRF41 cells by different methods (right panel): TOT, total RNA extraction; MNP, MNP enrichment method; and CENT, centrifugation enrichment method. The left and right panel electropherograms were obtained from running samples on an Agilent RNA ScreenTape and a High Sensitivity RNA ScreenTape, respectively. Electropherogram lanes are scaled to the highest intensity peak. Blue and red arrows next to the electropherograms represent host and bacterial ribosomal RNAs, respectively. (**b)** Percentage of total fragments mapped to the *E. piscicida* EtPo1 genome in enriched (MNP, CENT) and non-enriched (TOT) RNAs from infected BRF41 cells, and from control, broth-cultured bacteria (CTRL). (**c)** Principal component analysis of gene expression data in enriched (MNP, CENT), non-enriched (TOT), and control, broth-cultured (CTRL) *E. piscicida* RNAs. (**d)** Number of common DEGs (FDR < 0.001) between the MNP-enriched RNAs and centrifugation-enriched RNAs. (**e)** Map of the *E. piscicida* EtPo1 genome (light blue ring) showing the locations of all upregulated (red bars) and downregulated (blue bars) genes. The T3SS and T6SS loci are highlighted as green and light green bars, respectively, in the outer ring. (**f)** Heatmap of the normalized fragments per kilobase million (FPKM) expression values of genes in the T3SS (left) and T6SS (right) loci. Upregulated and downregulated genes are colored red and blue, respectively.

After verifying that magnetic capture was a viable approach to separate bacteria from lysates, we extracted RNA from magnetically recovered bacteria. Simultaneously, we infected cells with unlabeled bacteria and extracted bacterial RNA by two alternative methods for comparison. One method involved directly extracting total RNA from infected cells, while the other involved rapidly centrifuging bacteria from lysates before RNA extraction. The extracted RNAs were electrophoresed to visualize the relative amounts of host and bacterial RNAs with reference to RNAs mock mixed at different mass ratios (Fig. 3a). Bacterial RNA was undetectable in total RNA extracts but were detectable in extracts using the MNP and centrifugation-based differential lysis methods although there remained a considerable amount of host RNA (Fig. 3a). Recently, the *E. piscicida*-containing vacuole was found in association with the rough ER marker, calnexin (24), suggesting the bacteria recovered from digitonin-lysed hosts were partially surrounded by ribosome-studded membranes, which could account for the remaining host RNA in the extracts. Nonetheless, we prepared and sequenced strand-specific Illumina libraries after removing ribosomal RNA (Fig. S3) and mapped the fragments to an *E. piscicida* genome we assembled from Oxford Nanopore sequencing data (Fig. S4). Only about 0.04% of the total sequenced fragments on average from total extracted RNA could be mapped to the genome (Fig. 3b; Table S1). Conversely, the mapping rates were increased by at least an order of magnitude on average in MNP and centrifugation-enriched RNAs compared to total extracted RNAs (Fig. 3b). We anticipated that the mapping rate of the centrifugation-enriched RNAs would be lower than that of the MNP-enriched RNAs due to additional host RNA from co-pelleted organelles, but unexpectedly, it was not significantly different (Student’s unpaired *t*-test, *P* = 0.55). We ascribe this to the higher invasiveness of unlabeled bacteria, which results in an increased number of bacteria in lysates (Fig. 2e), thus the increased potential for bacterial RNA recovery by the centrifugation method. Nonetheless, when considering RNA enrichment and mapping rates based on the number of bacteria in primary lysates, the MNP method demonstrated the highest effectiveness compared to the other methods. Furthermore, the time between host lysis and RNA extraction by the MNP method (~7–8 minutes) is almost half that of the centrifugation method (~12–13 minutes), further minimizing temporal artifacts on gene expression analysis.

### Total RNA extracts lack sufficient information for differential gene expression analysis

Principal component analysis (PCA) of the gene expression data revealed that the total RNA extracts were distantly clustered from control, broth-cultured bacterial RNA samples and other enriched RNAs (Fig. 3c). We believe this was due to the extremely low number of mapped fragments and the concurrent low representation of genes in the total RNA extract data set (Fig. S5). Indeed, when we subsampled reads from sequencing saturated broth culture libraries, PCA showed an increasing divergence from the full set of reads as the number of mapped fragments decreased (Fig. S5). Consequently, we chose to focus on the enriched RNAs, which clustered closer to the control group for conducting differential gene expression analysis (Fig. 3c). To identify a core set of genes involved with intracellular infection, we set a stringent false discovery rate corrected *P*-value cutoff of 0.001 and focused on the common differentially expressed genes (DEGs) between the two enriched RNA groups. Leveraging information from both methods also allowed us to remove method-specific biases introduced on the gene expression data. About 9% (317 genes) of the *E. piscicida* genome was differentially expressed during intracellular infection of the host (Fig. 3d; Table S2), and consistent with previous findings of their essentiality during host infection, the T3SS and T6SS loci (9, 10, 26, 27) were hotspots of upregulated gene expression (Fig. 3e and f).

### The intracellular *E. piscicida* transcriptome reflects a mitochondrion-like energy generation program

**Fig 4:**
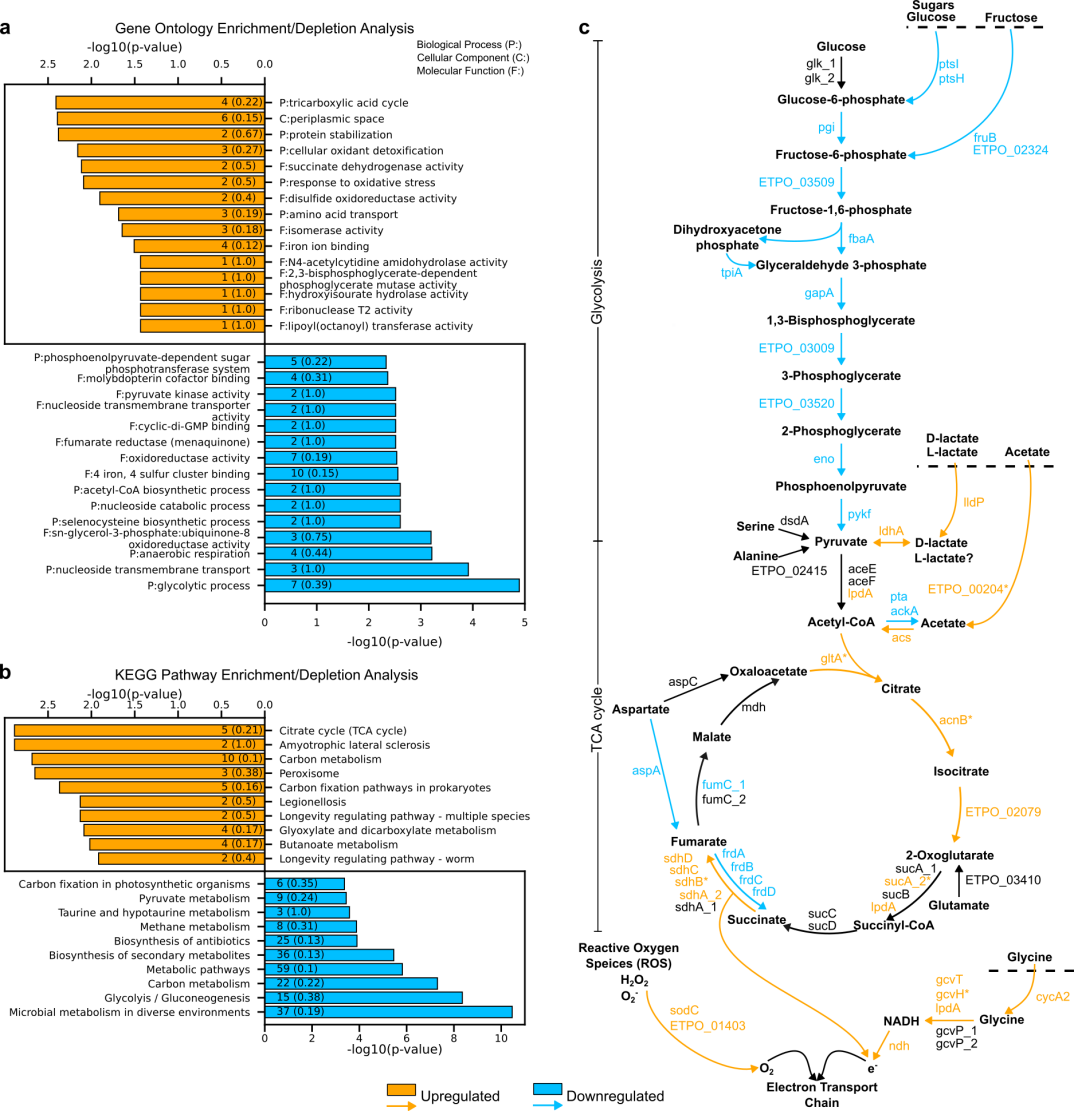
Bacterial sugar transport and glycolysis do not constitute the preferred carbon and energy source for *E. piscicida* during intracellular infection. (**a and b)** GO term (**a**) and KEGG pathway (**b**) enrichment analysis of DEGs in *E. piscicida* during intracellular infection of zebrafish fin fibroblasts. Orange and blue bars represent the -log10 *P* values for enriched annotations of upregulated and downregulated genes, respectively. The numbers on the bars represent the number of DEGs with the given annotation, and the numbers in parentheses represent the ratio of DEGs to all genes with the annotation. (**c)** Central carbon metabolism, metabolite transport, and energy generation pathways of *E. piscicida* during intracellular infection of zebrafish fibroblasts. Orange and blue arrows represent upregulated and downregulated processes, respectively. Black arrows represent unaltered processes. The genes involved with each process are labeled next to each arrow and are colored orange, blue, or black if they are significantly (FDR < 0.001) upregulated, significantly downregulated, or unaffected, respectively. DEGs with near significant (FDR < 0.05) changes in expression levels are marked with an asterisk. The corresponding arrow is colored if at least 60% of the genes involved with a process are differentially expressed. Black dotted lines represent bacterial membranes. All genes, gene products, and fold changes are listed in Table S7.

To gain a broad overview of the *E. piscicida* transcriptional landscape, we performed enrichment analyses on the Gene Ontology (GO) and Kyoto Encyclopedia of Genes and Genomes (KEGG) annotations of the upregulated and downregulated gene sets. These analyses revealed the common theme that upregulated genes were associated with the tricarboxylic acid (TCA) cycle and homeostasis of reactive oxygen species (ROS), while downregulated genes were associated with glycolysis and the phosphoenolpyruvate-dependent phosphotransferase system responsible for carbohydrate transport (Fig. 4a and b; Tables S3 to S6). Closer interrogation of the genes involved with glycolysis and the TCA cycle revealed that, indeed, while the majority of genes involved with glucose catabolism, and several with sugar import, were downregulated, many genes in the TCA cycle were conversely upregulated (Fig. 4c; Fig. S6; Table S7). This strongly suggested a major reshuffling of central carbon metabolism and energy generation during infection in which bacterial glycolysis does not generate the preferred carbon source for the TCA cycle.

To identify possible carbon sources for the activated TCA cycle, we systematically searched for upregulated genes that catalyze single reactions that flux metabolites directly into TCA intermediates. We found that the genes for acetyl-CoA synthetase (*acs*), which catalyzes the conversion of acetate to acetyl-CoA (28), and D-lactate dehydrogenase (*ldhA*), which catalyzes the interconversion of D-lactate and pyruvate (29), were upregulated (Table S7). Of note, the genes for an acetate uptake transporter (ETPO_00204), immediately tandemly downstream of *acs* (Table S7; Fig. S7) (28), and an L-lactate/D-lactate transporter (*lldP*) (30) were also upregulated. Excess production of lactate in eukaryotic cells as a result of hyperactive glucose metabolism, commonly referred to as the Warburg effect, is a well-known characteristic of proliferating cancer cells and infected cells (31). Recently, acetate has also been found to be produced during hyperactive glucose metabolism and its production is coupled to ROS (32), which may have some relationship to the host’s production of defensive ROS. While acetate import has a clear link to the bacterial TCA cycle through *acs*, the relationship between L-lactate uptake, D-lactate dehydrogenase activity, and the TCA cycle was less apparent as L-lactate is the predominant stereoisomer produced by vertebrates (33–35). Furthermore, we could not identify an L-lactate dehydrogenase coded within the *E. piscicida* genome. Notably, a predicted L-lactate utilization protein C (36) (ETPO_00341) is encoded about 2 kbp tandemly downstream of *lldP* though its expression was not significantly altered and its exact mechanisms of utilization are unknown (Fig. S7; Table S7).

Another curious observation was that while the expression levels of *aceE* and *aceF*, which encode the E1 and E2 components of the pyruvate dehydrogenase complex (PDHC) (37), respectively, were not significantly altered, there was a significant upregulation of *lpdA* encoding the E3 component and the PDHC transcriptional repressor *pdhR* upstream of these genes (Fig. S7; Table S7). The E3 component of the PDHC is shared between different 2-oxo acid dehydrogenase complexes (37, 38) and the glycine decarboxylase complex (39, 40) (GDC), important generators of NADH for the electron transport chain (ETC). The upregulation of *lpdA* and *pdhR* suggested a shift in complex preference for NADH production as *lpdA* is known to be expressed from an independent promoter to control the amount of E3 required by other complexes (37). The *E. piscicida* genome encodes enzymes for the oxoglutarate dehydrogenase complex, a key control point in the TCA cycle, and the GDC, which catabolizes glycine to produce NADH and one-carbon units for various metabolic processes. We found that several components of the GDC were upregulated and notably a glycine transporter, *cycA2* (41), was also upregulated. If glycine is abundantly accessible to intracellular *E. piscicida*, we can envision a faster and energetically cheaper flux of NADH into the ETC for energy production mediated by glycine transport and catabolism compared to glucose transport and glycolysis. Indeed, glycine catabolism and low glucose uptake were recently found to be characteristic of rapidly growing *Escherichia coli* in the early growth phase (42). Furthermore, *ndh* encoding the type II NADH:quinone oxidoreductase of complex I of the ETC was also upregulated in our data set further supporting this idea.

Put together, our interpretation of these data is that *E. piscicida* is bypassing sugar transport and glycolysis and actively importing host-derived, simple organic acids that can flux directly into its TCA cycle and ETC, which would accelerate oxidative phosphorylation for energy production. Oxidative phosphorylation is further intensified by the excess oxygen introduced by catalase- and superoxide dismutase-mediated detoxification of the host’s defensive ROS. To envision this in another light, some aspects of this interpretation curiously mirror how mitochondria generate energy by delegating glycolysis and catabolism of complex macromolecules to the “host” while importing pyruvate and other simple organic acids to directly fuel its TCA cycle and ETC.

### Excludons are involved with intracellular acclimatization

When visualizing the RNA mapping of our data set, we observed varying levels of transcripts that aligned antisense to coding regions and resembled those arising from overlapping transcription of convergent/divergent gene pairs. The antisense transcript (AT) levels varied between broth culture and infection groups (MNP and centrifugation-enriched RNAs) hinting at involvement of excludons during intracellular acclimatization. To identify putative excludons involved with intracellular acclimatization in our data set, we used two different approaches.

**Fig 5:**
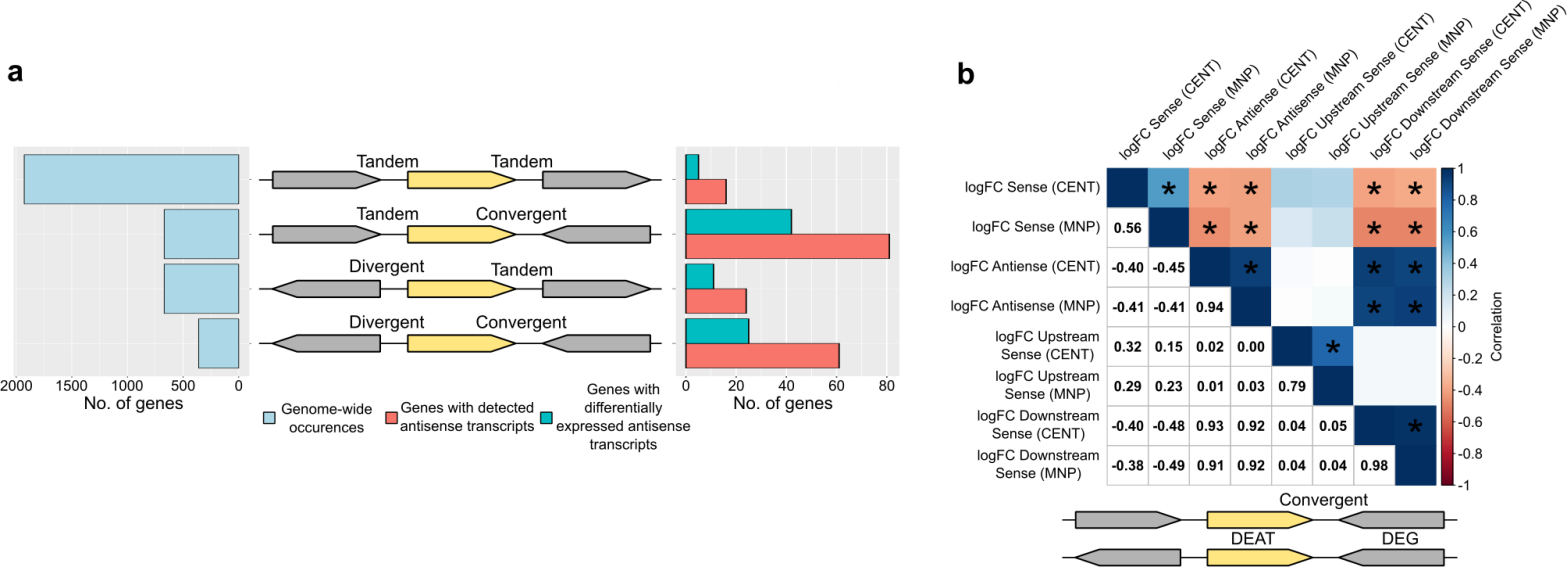
Orientational contexts of genes on the *E. piscicida* chromosome and correlation analysis of AT and ST fold changes of responder genes in convergent excludons (see text). (**a)** Orientational contexts for each gene and its upstream and downstream neighbors in the *E. piscicida* genome, and the number of genes with detectable antisense transcripts and differentially expressed antisense transcripts in each context. (**b)** For responder genes in convergent excludons, a correlation matrix of the Pearson’s correlations between fold changes of their sense, antisense, downstream counterparts’ sense, and upstream counterparts’ sense transcripts. Correlations are scaled to color and directly displayed in the upper and lower triangles, respectively. Significant correlations (*P <* 0.05) are marked by an asterisk in the upper triangle.

In the first approach, for all genes with detectable ATs, we correlated the AT fold changes with the sense transcript (ST) fold changes of their upstream or downstream counterparts and narrowed these down to those with the highest correlation by filtering fold changes based on significance. A high correlation would suggest that the ATs and STs of their counterparts are expressed at the same level, and thus possibly encoded on the same RNA. Furthermore, excludons predicted by this approach could identify those involved with intracellular acclimatization because the DEGs and differentially expressed antisense transcripts (DEATs) used for prediction are identified specifically from an intracellular population of bacteria. First, we categorized the possible orientational contexts for a given gene and its immediate upstream and downstream neighbors and tallied the genome-wide occurrences of each context (Fig. 5a). Most genes are in a tandem-tandem context as is expected in the operonic nature of bacterial genomes. Profiling the genes with detectable ATs revealed that the majority of their downstream counterparts were convergent (Fig. 5a). Furthermore, of the genes with DEATs, the majority were also in a convergent pair. When we correlated the AT fold changes with the ST fold changes of upstream or downstream counterparts, we generally saw strong positive correlations in convergent gene pairs, while there was little to none in divergent gene pairs (Fig. S8). The positive correlation was strongest (0.91–0.93) for genes in convergent pairs when both their ATs and their downstream counterparts’ STs were significantly differentially expressed (Fig. 5b). Notably, the negative correlation between STs and ATs (−0.40 to −0.45) also became stronger in this scenario (Fig. 5b) contrasting the weak correlation observed on a genome-wide comparison (−0.10 to −0.21) (Fig. S8). We termed these 30 genes (Table S8), i.e., genes in which their DEAT fold changes were strongly correlated with their downstream convergent counterparts’ differentially expressed ST fold changes, as responder genes in convergent excludons. We use the term “responder” here as DEATs could be detected from these genes while AT from their counterparts, if any, were not differentially expressed. It is worth mentioning that we were also able to identify three genes in divergent pairs in which the AT and the ST of their upstream gene were differentially expressed (Table S9; Fig. S9). However, these were insufficient data points to perform correlation analysis.

**Fig 6:**
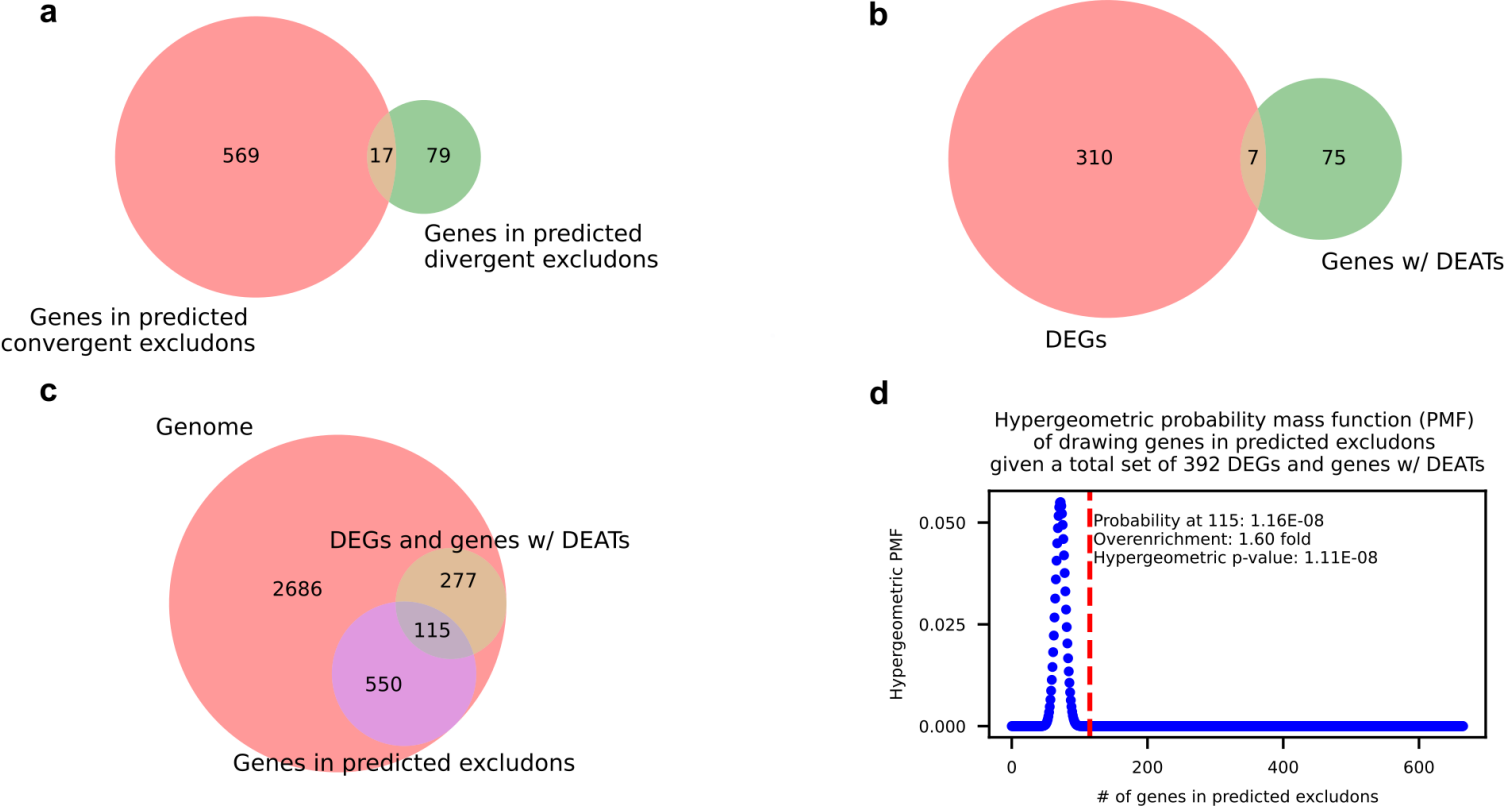
Genes in predicted excludons are enriched in the set of genes with perturbed expression during intracellular infection. (**a)** Venn diagram of the sets of genes in convergent and divergent excludons predicted by identification of RNA read fragments mapping across intergenic regions. (**b)** Venn diagram of the set of all genes with significantly perturbed expression during intracellular infection (the sets of DEGs and genes with DEATs). (**c)** Venn diagram of the set of all genes in predicted excludons and the set of all genes with significantly perturbed expression during intracellular infection in the *E. piscicida* genome. (**d)** Hypergeometric probability mass function of drawing any number of the 665 genes in predicted excludons from a total set of 392 genes with perturbed expression during intracellular infection. The dotted red line marks the PMF when 115 genes are drawn.

In our second approach, we directly detected transcript fragments that encoded both sense and antisense sequences of genes in convergent and divergent pairs by extracting read fragments that mapped across the intergenic regions. We focused on sequencing data from broth-cultured bacteria as they were sequenced deeply enough to cover most of the genome. Due to the nature of the short read data, our ability to confidently predict overlapping transcription was restricted to gene pairs with intergenic distances less than 300 bp, as only about 10% of the mapped fragments had lengths that exceeded this threshold (Fig. S10). We found that 293 of 422 (69%) and 48 of 268 (18%) convergent and divergent gene pairs with intergenic distances less than 300 bp, respectively, showed overlapping transcription of coding sequences (Fig. S10). In total, by this approach, there were 665 genes that were predicted to be members of a convergent or divergent excludon or both (Fig. 6a). To assess their significance in intracellular acclimatization, we conducted a hypergeometric test for their enrichment in the set of all genes with perturbed expression during intracellular infection, i.e., the set of all DEGs and genes with DEATs (Fig. 6b). One hundred fifteen genes within predicted excludons were found in the set of 392 genes with perturbed expression (Fig. 6c), and the hypergeometric test revealed that they were significantly enriched by 1.6-fold compared to the expected background (Fig. 6d).

**Fig 7:**
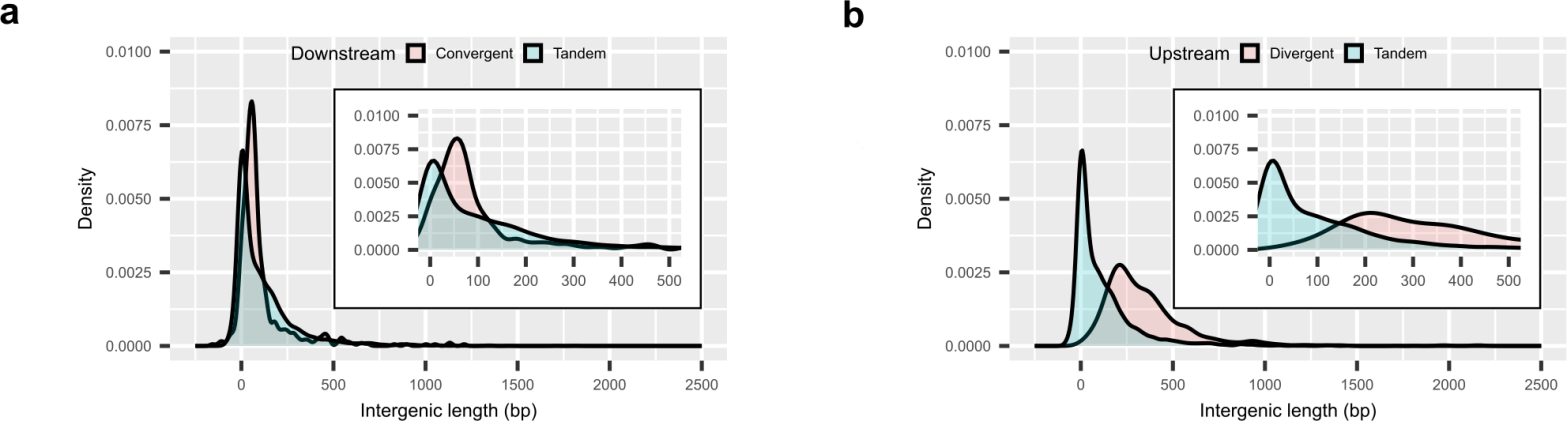
Intergenic distances between convergent and divergent gene pairs in the *E. piscicida* genome. (**a and b)** Density plot of downstream (**a**) and upstream (**b**) intergenic distances between gene pairs. Insets show the range from 0 to 500 bp.

**Fig 8:**
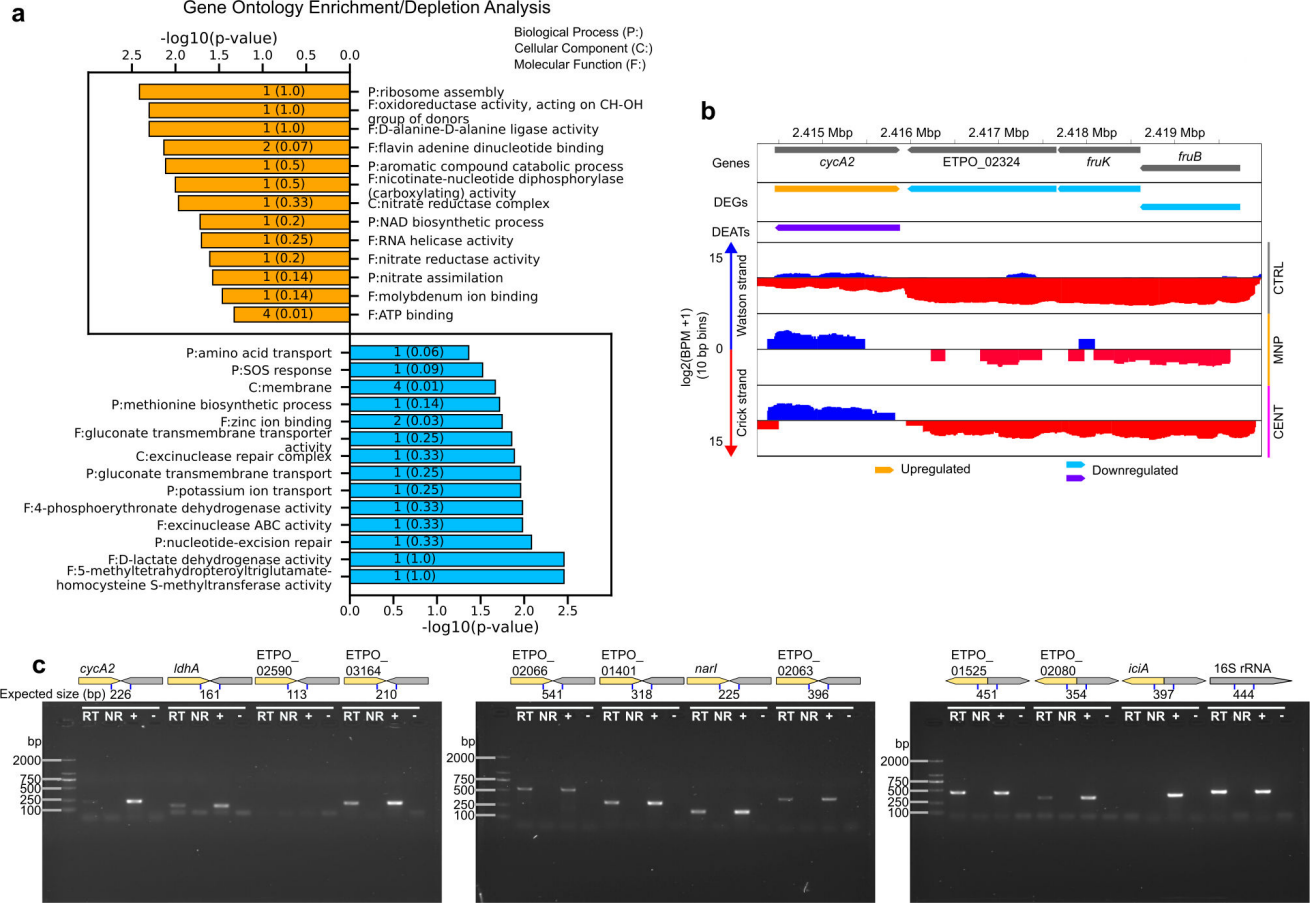
Potential roles and RT-PCR validation of predicted excludons. (a) GO enrichment analysis of responder genes in convergent excludons. Orange and blue bars represent enriched annotations for upregulated and downregulated antisense transcripts, respectively. Numbers on the bars represent the number of genes with the given annotation, and numbers in parentheses represent the ratio of genes to all genes with the annotation. (b) Representative responder gene in convergent excludon, *cycA2*, in a convergent gene pair with ETPO_02324. The first (upper) panel shows the positions and orientations of genes. The second panel shows DEGs: orange, upregulated; blue, downregulated. The third panel shows DEATs: purple, downregulated. The bottom three panels show the mean log2 fragments per bins per million mapped reads in 10 bp bins mapped to the Watson (blue) and Crick (red) strands in control bacterial RNAs (gray bar), MNP-enriched RNAs (orange bar), and centrifuge-enriched RNAs (pink bar). (c) RNA from broth-cultured bacteria was used for RT-PCR confirmation of overlapping transcription of predicted convergent and divergent excludons using primers designed within 100 bp upstream of the stop codons or 100 bp downstream of the start codons for convergent or divergent gene pairs, respectively. An internal fragment of the 16S rRNA gene was used as an RT control. Templates in PCR reaction: RT, RT reaction with enzyme; NR, RT reaction with no enzyme; +, 1 ng gDNA; and −, sterile water.

Taken together, the findings from the two approaches suggest that in *E. piscicida,* genome-wide PAT and excludons are more common in convergent gene pairs than in divergent pairs and that excludons are involved with intracellular acclimatization. Generally, the intergenic distance in divergent pairs is larger than in convergent pairs in *E. piscicida* (Fig. 7), thus the chance for overlapping transcription within coding sequences may be lower. Convergent excludons were also more common in another Gammaproteobacterium, *Dickeya dadantii* (43). We conducted GO term enrichment analysis of the 30 responder genes in convergent excludons identified from our first approach to gain an overview of the roles that excludons and DEATs may play during intracellular infection. We chose the genes in these excludons because both members of the excludons had significant differential expression of ATs and STs and are more likely to be directly involved with intracellular acclimatization, while the excludons predicted from our second approach may involve instances where only one member exhibits perturbed expression during intracellular infection. The analysis highlighted certain similarities consistent with the GO analysis of DEGs. Terms for amino acid transport and D-lactate dehydrogenase activity were enriched in genes with downregulated ATs and upregulated STs, and nitrate assimilation was enriched in upregulated ATs and downregulated STs (Fig. 8a and 4a; Tables S3, S4, S10, and S11). Most prominently, the amino acid transporter, *cycA2*, is in a convergent excludon with ETPO_02324, one of the fructose-specific PTS components, establishing a direct link to the reorganization of central carbon metabolism through the switch in metabolite preference (Fig. 8b and 4c). Furthermore, *ldhA* is in a convergent excludon with ETPO_01809, a putative glycogen synthesis protein, and *narI*, a nitrate reductase gamma subunit, is in a convergent excludon with *sodC* (Fig. S11) further strengthening the association of excludons with the switch to intracellular acclimatization. We note that the involvement of excludons was not limited only to convergent pairs but also extended to divergent pairs as exemplified by *gapA*, a central glycolytic enzyme, existing as a divergent excludon with ETPO_01525 (Fig. S9), a peptide-methionine (R)-S-oxide reductase involved in oxidative stress resistance. Finally, to verify our approach for predicting excludons, we also confirmed the presence of overlapping RNA transcription of coding sequences in seven of eight predicted convergent excludons and two of three predicted divergent excludons by reverse transcription polymerase chain reaction (RT-PCR) (Fig. 8c).

## DISCUSSION

Obtaining sufficient sequencing data for comprehensive gene expression analysis has been a major hurdle in profiling the transcriptional landscape of intracellular bacterial pathogens during intracellular infection of host cells due to overwhelmingly excessive host RNAs in extracts (12). There have been several landmark breakthroughs in the enrichment of bacterial RNA (44–49), yet they rely on large-scale experiments to recover sufficient bacterial RNA (48) or restrictive probe sets in hybridization-based enrichment approaches (49). Here, we developed a quick and simple method to label an intracellular bacterium, *E. piscicida*, with MNPs prior to infection of host cells. By magnetically reisolating bacteria from lysates, we could enrich bacterial RNA in extracts, thereby allowing us to profile both sense and antisense transcription through sequencing in an approach that is small scale, inexpensive, and able to capture all transcripts. We envision coupling the magnetic separation of bacteria to the lysis of host cells on microfluidic devices as a promising approach to single-cell sequencing of intracellular pathogens, another frontier of difficulty in studying these pathogens.

Our analysis revealed that *E. piscicida* activates a hyperactive energy generation program during intracellular infection of host cells. This program can be conceptualized as one that skips over the import and catabolism of sugars and instead preferentially imports and catabolizes simple organic acids that require a shorter flow of their electrons to acceptors in the ETC. While mitochondria employ a similar program to generate energy for the eukaryotic “host,” in *E. piscicida*, the energy generated is for itself, and the molecules it prefers to import seem to differ. Mitochondria lack glycolytic enzymes, but *E. piscicida* likely retains its glycolytic pathway for metabolic flexibility due to the diverse environments it inhabits during its non-infective cycle (7) and may activate this program only when conditions are appropriate. This program or its derivatives may be common to other intracellular bacteria as well. In another related facultative, intracellular Gammaproteobacterium, *Salmonella enterica*, genes involved in glycolysis have also been observed to be repressed up to 85-fold during intracellular infection of macrophages (48). Similarly, *Yersinia pestisp*, a facultative, intracellular Gammaroteobacterium, downregulates the expression of glycolytic genes while upregulating expression of genes in the TCA cycle during replication in macrophages (50). Additionally, in an obligate, intracellular Alphaproteobacterium, *Rickettsia typhi*, there is evidence for a functioning TCA cycle but glycolytic enzyme activities are all but undetectable (51, 52). As *E. piscicida*, other intracellular Proteobacteria pathogens, and mitochondria appear to employ a similar energy generation program, this suggests a potentially conserved mechanism for optimized energy acquisition from hosts centered around the TCA cycle, likely derived from a common ancestor.

We found that several genes involved with the switch to intracellular acclimatization were members of convergent excludons, suggesting that nutrient transport, central carbon metabolism, ROS homeostasis, and virulence in *E. piscicida* are linked in a complex regulatory network through PAT and excludons. We note that while our findings suggest only a general involvement of excludons with intracellular acclimatization, the extent to which each excludon plays a role remains an open question. Future studies characterizing transcript starts and ends in order to determine excludon/operon structures can guide gene knockout/manipulation experiments to corroborate phenotypes with the predicted functions of genes in excludons as they relate to intracellular acclimatization. Furthermore, correlating RNA and protein expression levels by transcriptomics and proteomics specifically for genes in excludons may shed some light on how excludons regulate protein expression. Studies on the rat (53) and human (54) mitochondrial RNAs have revealed the existence of antisense RNAs arising from the overlapping transcription of convergent gene pairs, suggesting that this mode of gene expression regulation is vital in small genomes, ranging from endosymbionts to intracellular pathogens.

In summary, we developed a novel method employing magnetic nanoparticles to explore the transcriptome of an aquatic Gammaproteobacterium, *Edwardsiella piscicida*, during intracellular infection of host cells. Our findings reveal that *E. piscicida*’s energy generation program mirrors that of mitochondria—energy powerhouses with a hyperactive TCA cycle devoid of glycolytic processes. Notably, several implicated genes are members of excludons—gene pairs antagonistically co-regulated by overlapping antisense transcription. Other intracellular Proteobacterial pathogens appear to adopt a similar mitochondrion-like energy generation program, suggesting a potentially conserved strategy for optimized energy acquisition from hosts centered around the TCA cycle.

## MATERIALS AND METHODS

### Media, strains, and culture conditions

*Edwardsiella tarda* strain EtPo1, originally isolated by I. Hirono from the liver of olive flounder (*Paralichthys olivaceus*) was reclassified to *E. piscicida* based on Oxford Nanopore sequencing data and average nucleotide identities to reference genome sequences of representative *Edwardsiella* spp. *E. piscicida* was routinely cultured at 30°C in tryptic soy broth (TSB) (Formedium) on a shaking incubator set at 170 rpm or on tryptic soy agar (TSA) (Millipore) plates. Zebrafish, *Danio rerio*, fin fibroblasts, BRF41 (RCB0804), were purchased from the RIKEN BRC Cell Bank and cultured at 33°C in Leibovitz’ L-15 medium (ThermoFisher) supplemented with 10 mM HEPES (ThermoFisher) and 15% fetal bovine serum (referred to as complete L-15 medium hereafter).

### DNA extraction, Nanopore sequencing, genome assembly, and functional annotation

*E. piscicida* was grown to an OD_600_ of 1.0 (approx. 1 × 10^9^ CFU/mL), and 1 mL of the bacterial culture was taken for DNA extraction using the Nanobind Tissue Big DNA Kit (Circulomics) following the manufacturer’s protocol developed for Gram-negative bacteria (EXT-GNH-001). Sequencing libraries were prepared using the Nanopore Ligation Sequencing kit (SQK-LSK110, ONT) and sequenced on a Flongle flow cell (FLO-FLG001, ONT) on the MinION Mk1C (ONT) system. The MinKNOW software controller (version 22.05.8) was used to collect signal data in which basecalling was done using Guppy (version 6.1.5). NanoLyse (55) (version 1.2.1) and PoreChop (v. 0.2.4) were used to remove control lambda sequences and sequencing adapters, respectively, from the read files. NanoFilt (v. 2.8.0) was used to filter and retain reads with an average *Q* score greater than 12, and the filtered reads were assembled using the Flye assembler (version 2.6) with an estimated genome size parameter of 3.7 Mbp. The primary assembly was polished by mapping the Q12 filtered reads using minimap2 (56) (version 2.24-r1122) and polished by Racon (57) (version 1.1.1). Polishing was reiterated once more using the filtered Q12 reads. Subsequently, polishing of coding sequences was undertaken using short-read Illumina sequences of RNA extracted from broth-cultured bacteria (see below). Short-read sequences were mapped to the polished assembly using bowtie2 (58) (version 2.3.2) and polished using Pilon (59) (version 1.24). Short-read polishing was reiterated once more to produce the final assembly. The final assembly was imported into Geneious Prime (2022.2.2 release) and circularized at the start codon of the *dnaA* gene as determined by BLAST of the *Escherichia coli dnaA* gene (U00096.3:3882326–3883729) on the assembly. The completeness of the assembly was assessed with Benchmarking Universal Single-Copy Orthologs (60) (version 5.4.7) using the enterobacterales_odb10 lineage. Average nucleotide identities in comparison to representative reference genome sequences of *Edwardsiella* spp*.* were computed using fastANI (61) (version 1.33). The reference GenBank accession numbers of *E. piscicida* 18EpOKYJ, *Edwardsiella anguillarum* ET080813, *Edwardsiella ictaluri* S07-698, *E. tarda* KC-Pc-HB1, and *Edwardsiella hoshinae* FDAARGOS_940 are GCF_021733145.1, GCF_000264765.2, GCF_003074995.2, GCF_002504285.1, and GCF_016026395.1, respectively. Gene prediction was done using Prokka (62) (version 1.12), and Blast2GO Basic (63) (version 6.0) was used to determine putative gene functions and GO annotations. KEGG pathway annotations were predicted using eggNOG mapper (64) (version 2.1.9).

### Labeling of *E. piscicida* with magnetic nanoparticles

The attachment of nanoparticles to the surfaces of Gram-positive and Gram-negative bacteria has previously been attributed to the zeta-potentials of the bacterial and nanoparticle surface and can be explained by the DLVO theory, which models the aggregation behavior of colloidal particles in a liquid medium (19, 20). Labeling was carried out with carboxyl-coated superparamagnetic nanoparticles (referred to as MNPs hereafter) as previously described (21) with minor modifications. Specifically, monodisperse, spherical MNPs prepared by sequentially coating layers of magnetite and polystyrene onto core polystyrene particles were employed in place of irregularly shaped MNPs, and the carboxyl functional groups were not activated by carbodiimide. MNPs (CM-025-10H, Spherotech) were supplied from the manufacturer in a stock solution of 10 mg/mL and were reported to have a mean diameter of 250 nm as determined by dynamic light scattering analysis and a density of 2.73 g/cm^3^. Given the density, diameter, and mass concentration of the nanoparticles, we can estimate the number of nanoparticles in a given volume by the formula provided by the manufacturer


$$N=\left(6W÷\pi PD^{3}\right)\times 10^{12}$$

where *N* is the number of nanoparticles, *W* is the mass of nanoparticles in a given volume in grams, *P* is the density of the nanoparticles in g/cm^3^, and *D* is the diameter of the nanoparticles in micrometers. This formula estimates the volume and mass of a single nanoparticle by the diameter and density parameters and approximates the total number of particles in a given mass by dividing the total mass of nanoparticles by the estimated mass of a single nanoparticle. In 1 mL of the stock solution, there are approximately 447.7 × 10^9^ MNPs. The stock solution was diluted to 1 mg/mL in 1 mL of phosphate-buffered saline (PBS) (ThermoFisher) in a microfuge tube and washed twice with equal volumes of PBS by magnetic separation of the MNPs on a DynaMag-2 magnetic rack (Applied Biosystems). Washed MNPs were pulse sonicated on ice for 5 s intervals with 25 s pauses for a total duration of 5 minutes using a TomyUltrasonic Disruptor UD-211 set at 40% power output. The sonicated MNP solution was briefly centrifuged at 1,000 *g* for 30 s, and 125 µL fractions of the supernatant were carefully transferred to new microfuge tubes and the volumes were brought up to 500 µL with PBS. Assuming a 10% loss of MNPs due to the size fractionation process, there are approximately 5 × 10^9^ MNPs in these suspensions. Size-fractionated MNPs were taken for size distribution analysis by TEM (see below). *E. piscicida* grown to an OD_600_ of 1.0 (approx. 1 × 10^9^ CFU/mL) in 5 mL of TSB were washed twice in equal volumes of PBS by centrifugation at 5,000 *g* for 5 minutes and resuspended in 2.5 mL of PBS to concentrate the bacteria. Five hundred microliters of the bacterial suspension (approx. 1 × 10^9^ CFU) was added to 500 µL of the size-fractionated MNP suspension and incubated at 30°C for 30 minutes on a rotator set at 12 rpm. After incubation, the microfuge tube was spun down for 5 s on a mini centrifuge and placed on a magnetic rack for at least 2 minutes to capture MNP-labeled *E. piscicida*. The supernatant with non-labeled bacteria was carefully removed, 1 mL of PBS was immediately added to the tube, and the MNP-labeled bacteria were resuspended by gentle pipetting. The MNP-labeled bacteria were washed once again on the magnetic rack before finally resuspending in 1 mL of PBS for serial dilution and plating on TSA to count the number of recovered CFUs, 1 mL of 4% paraformaldehyde (PFA) in PBS for fixing prior to imaging by TEM, or 1 mL of complete L-15 medium for infection experiments.

### Transmission electron microscopy

Size-fractionated MNPs were washed twice with 500 µL of sterile distilled water (SDW) using a magnetic rack and resuspended in 500 µL of SDW. Five microliters of the MNP suspension was applied to glow discharged, 200-mesh formvar/carbon-supported copper grids (Sigma Aldrich) and allowed to air dry. Grids were imaged under a JEM-1400 TEM (JEOL) at 120 kV using the built-in CCD camera. Images were used for particle size distribution analysis in ImageJ (Fiji) by manual measurement using the measure tool. MNP-labeled bacteria resuspended in 4% PFA were fixed on a rotator for 30 minutes at room temperature and washed twice with 500 µL of SDW using a magnetic rack before resuspension in 500 µL of SDW. The fixed bacterial suspension was diluted, and 5 µL of the suspension was applied to glow-discharged, 200-mesh formvar/carbon-supported copper grids (Sigma Aldrich) and allowed to air dry. Grids were imaged under a JEM-1400 TEM at 120 kV using the built-in CCD camera. Images of single cells were used to count the number of MNPs labeled to bacteria.

### MNP effect on *E. piscicida* viability

To test the effect of MNP labeling on the viability of *E. piscicida*, we prepared size-fractionated MNPs and *E. piscicida* in triplicate as described in the labeling procedure. The MNPs and bacteria were mixed and incubated at 30°C on a rotator set at 12 rpm for 2 h. A 50 µL sample was taken from the tube just after mixing (time 0) and every 30 minutes for serial dilution and plating on TSA to count the number of viable CFUs the next day. As a control, *E. piscicida* were incubated with 500 µL of PBS and similarly assayed for viability.

### Subcellular fractionation of zebrafish fibroblasts and western blotting

Subcellular fractionation of BRF41 cells was carried out as previously described for mammalian cells (23) with minor modifications. Briefly, 2 × 10^5^ BRF41 cells were seeded in the wells of a 12-well plate 1 day prior to subcellular fractionation. On the day of fractionation, cells were trypsinized in 500 µL of Trypsin-EDTA (0.25%) solution (Gibco) and transferred to microfuge tubes containing 500 µL of complete L-15 medium to quench the trypsin solution. The cells were centrifuged at 100 *g* at 4°C for 5 minutes, the supernatant was removed, and the cells were gently washed with 1 mL of ice-cold PBS. The cells were pelleted once again by centrifugation and the supernatant was aspirated. One hundred microliters of ice-cold, freshly prepared, 0.22-µm filtered lysis buffer (150 mM NaCl, 50 mM HEPES [N-2-hydroxyethylpiperazine-N-2-ethane sulfonic acid] [Gibco], pH 7.4) containing digitonin (Sigma Aldrich) at final concentrations of 0, 1, 5, 10, 25, 50, 100, 150, and 200 µg/mL and ProteoGuard EDTA-free protease inhibitor cocktail (Clontech) at a 1× concentration was added to the tubes separately, and the cells were gently resuspended by pipetting. The cells were incubated at 4°C for 5 minutes and then centrifuged at 2,000 *g* for 5 minutes to pellet intracellular, membrane-bound organelles. The supernatant containing the cytosolic protein fraction was aspirated and stored at −20°C until protein concentration quantification and western blot analysis. The pellet was then resuspended by vortexing in 100 µL of ice-cold, freshly prepared, 0.22-µm filtered lysis buffer containing 1% NP-40 and ProteoGuard EDTA-free protease inhibitor cocktail at a 1× concentration and incubated on ice for 30 minutes. The tube was then centrifuged at 7,000 × *g,* and the supernatant containing the membrane-bound organelle protein fraction was aspirated and stored at −20°C. As a control, cells from one well of the 12-well plate were similarly trypsinized and quenched but resuspended in 100 µL of ice-cold, freshly prepared, 0.22-µm filtered RIPA buffer (150 mM NaCl, 5 mM EDTA, 50 mM Tris, 1% NP-40, 0.5% sodium deoxycholate, and 0.1% SDS) containing ProteoGuard EDTA-free protease inhibitor cocktail at a 1× concentration, incubated on ice for 30 minutes, centrifuged at 7,000 *g,* and the supernatant was aspirated to collect whole cell lysates. Protein concentrations were determined using the Takara BCA Protein Assay Kit (Takara). Equal fractions (30 µL) were boiled at 95°C for 5 minutes in standard SDS-PAGE sample loading buffer containing 10% 2-mercaptoethanol and resolved on a 4%–10% SDS-PAGE gel. Proteins were transferred onto Immun-Blot LF PVDF membranes (Bio-Rad). Membranes were blocked in bovine serum albumin (BSA) diluent/blocking solution (SeraCare) diluted to 1% BSA in PBS for 1 h at room temperature. Membranes were then washed thrice with PBST (PBS supplemented with 0.1% Tween-20) for 5 minutes each time. Membranes were incubated with anti-HSPA5 (AP5041c, Abcepta) primary antibodies diluted at a 1:1,000 (vol/vol) dilution in Western BloT Immuno Booster (Takara) Solution 1 for 1 h at room temperature. The membranes were washed again thrice with PBST for 5 minutes each time and then incubated with anti-rabbit IgG H&L, Alexa Fluor 790-conjugated (ab175781, Abcam) secondary antibodies diluted at a 1:10,000 (vol/vol) dilution in Western BloT Immuno Booster Solution 2 for 1 h at room temperature. The membranes were washed again thrice in PBST for 5 minutes each time before imaging on an Odyssey Fc imaging system (LI-COR). The membranes were stripped by incubating in EzReprobe (Atto) with gentle rocking for 20 minutes at room temperature and washed thrice with PBST for 5 minutes each time. The membranes were blocked once again as above, washed thrice in PBST, and incubated with anti-GAPDH (10494-1-AP, Proteintech) primary antibodies diluted at a 1:1,000 (vol/vol) dilution in Western BloT Immuno Booster Solution 1 for 1 h at room temperature. Washing, secondary antibody incubation, washing, and imaging were performed similarly as above.

### Establishment of intracellular infection model

We primarily established an intracellular infection model of *E. piscicida* in BRF41 cells by applying a gentamicin protection assay (25). 1 × 10^5^ BRF41 cells were seeded in the wells of a 24-well plate 1 day prior to infection. On the day of infection, *E. piscicida* cultures grown to an OD_600_ of 1.0 were washed twice with equal volumes of PBS by centrifugation, resuspended, diluted in complete L-15 medium to achieve an MOI of 50 in 100 µL, and added to the wells of the cultured cells in triplicate. The plates were centrifuged at 600 *g* for 10 minutes to promote bacterial adhesion and incubated at 33°C for 90 minutes to allow intracellular invasion. The wells were then washed three times with PBS, complete L-15 medium with or without 100 µg/mL gentamicin (Gibco) was added to the wells, and the plates were incubated for 3 h at 33°C to eliminate non-invaded bacteria. The wells were then washed three times with PBS and 500 µL of ice-cold, freshly prepared, 0.22-µm filtered, 25 µg/mL digitonin lysis buffer was added to each well and incubated for 5 minutes to lyse the BRF41 cells. The lysate was gently pipetted in the wells, transferred to a microfuge tube, serially diluted, and plated on TSA to count the number of surviving intracellular CFUs the next day.

### Infection of BRF41 cells with MNP-labeled bacteria and magnetic re-isolation of bacteria

For the infection of BRF41 cells with MNP-labeled bacteria, 1 × 10^5^ BRF41 cells were seeded in the wells of a 24-well plate 1 day prior to infection. On the day of infection, MNP-labeled bacteria prepared and resuspended in complete L-15 medium as described in the labeling procedure were diluted in complete L-15 medium to achieve MOIs of 50, 100, and 200 in 100 µL and added to the wells in triplicate. Intracellular infection was achieved using a gentamicin invasion assay as described above. The BRF41 lysate was gently pipetted into the wells, transferred to a microfuge tube, and 100 µL was taken, serially diluted, and plated on TSA to count the number of surviving intracellular CFUs the next day. To determine the ability to magnetically separate the MNP-labeled bacteria from the lysate, the microfuge tube was then placed on a magnetic rack for at least 2 minutes to capture MNP-labeled bacteria, the supernatant was carefully removed and 400 µL of PBS was added to resuspend the bacteria. The resuspended bacteria were then serially diluted and plated on TSA to count the number of CFUs remaining after magnetic capture the next day. The percentage of MNP-labeled *E. piscicida* CFUs remaining after magnetic capture was calculated as the total CFU in 400 µL of suspension after magnetic capture divided by the total CFU in 400 µL of lysate before magnetic capture multiplied by 100. As a control, infection with non-labeled bacteria and magnetic re-isolation were performed similarly.

### RNA extraction

RNA was extracted from BRF41 cells by TRIzol (Invitrogen) following the manufacturer’s instructions or from *E. piscicida* by homogenization in TRIzol with 0.1 mm zirconia/silica beads (Tomy) on a Precellys 24 homogenizer (Bertin Technologies) at 6,500 rpm for two 23-s cycles before the addition of chloroform and following the standard TRIzol procedure. All RNA samples were DNase I-treated (Takara) and repurified using a Monarch RNA Clean Up Kit (NEB), following the manufacturer’s instructions. Three methods were employed to recover bacterial RNA from infected cells in this study: (i) magnetic capture of MNP-labeled bacteria from lysed cells before RNA extraction; (ii) centrifugation to separate bacteria from lysed cells before RNA extraction; and (iii) direct, total RNA extraction from infected cells. For all methods, 5 × 10^5^ BRF41 cells seeded in the wells of 6-well plates were used for infection experiments. For the first method, cells were infected with MNP-labeled bacteria at an MOI of 50 in triplicate and re-isolated as described above with slight modifications. BRF41 cells were lysed in 1 mL of ice-cold 25 µg/mL digitonin lysis buffer, and then bacteria were magnetically captured with a magnetic rack and resuspended in 0.5 mL of ice-cold TRIzol for immediate homogenization and RNA extraction. For the second method, cells were infected with non-labeled bacteria at an MOI of 50 in triplicate. Cells were lysed in 1 mL of ice-cold 25 µg/mL digitonin lysis buffer, and bacteria were separated from the lysate by centrifugation at 5,000 × *g* for 5 minutes. The supernatant was removed, the pellet was resuspended in 0.5 mL of ice-cold TRIzol, and immediately homogenized for RNA extraction. For the third method, cells were infected with non-labeled bacteria at an MOI of 50 in triplicate. Cells were directly lysed in the plate in 1 mL of TRIzol, and RNA was immediately extracted. For the third method, one sample was lost during the RNA extraction procedure, but the loss had no effect on the findings of the research. As a control to compare gene expression, RNA was extracted from the same broth culture that was used to prepare MNP-labeled and unlabeled bacteria. As a reference to compare the relative amounts of bacterial and host RNA in the samples obtained from the three methods, separately purified BRF41 and *E. piscicida* RNA were mock mixed at host-to-bacteria mass ratios of 1:1, 2:1, 4:1, and 8:1. Specifically, 1 µL of 50 ng/µL *E. piscicida* RNA was added to 1 µL of 50, 100, 200, or 400 ng/µL BRF41 RNA. RNA samples were analyzed on an Agilent 2200 TapeStation system with the RNA ScreenTape reagents or High Sensitivity RNA ScreenTape reagents.

### cDNA library preparation and sequencing

Ribosomal RNA (rRNA) in the samples was depleted using the NEBNext rRNA Depletion Kit (Bacteria) (NEB) and cleaned up using Agencourt RNAClean XP Beads (Beckman Coulter). Strand-specific sequencing libraries were prepared using an Illumina Stranded mRNA Prep Kit (Illumina) following the manufacturer’s instructions with minor modifications. rRNA-depleted samples in 4.25 µL of nuclease-free water were mixed directly with 4.25 µL of the Elution, Prime, Fragment 3HC Mix (EPH3) solution, and fragmented/denatured at 94°C for 5 minutes. The remaining steps were performed according to the manufacturer’s instructions using half volumes of reagents, except the final cDNA elution step. The sizes and concentrations of the libraries were determined on an Agilent 2100 Tapestation using a D1000 ScreenTape. Libraries were pooled and sequenced on an Illumina Hiseq X system.

### RNA sequencing analysis

Reads were trimmed using Trimmomatic (65) (version 0.38) with options (ILLUMINACLIP:NexteraPE-PE.fa:2:30:10:2:keepBothReads HEADCROP:1 SLIDINGWINDOW:4:20 MINLEN:50) and imported into the CLC Genomics Workbench (version 8.0.1) software. Reads were aligned to coding sequences of the EtPo1 genome assembled from Oxford Nanopore sequencing data, and the quantile-normalized, log2-transformed fragments per kilobase per million mapped reads plus 1 (log2 FPKM + 1) values were used to generate PCA plots. Libraries prepared from total RNAs extracted from infected cells showed a large divergence from other prepared libraries and were not used in further analyses. Differential gene expression analysis was done using the empirical analysis of the DGE method (also incorporated in the EdgeR Bioconductor package) (66) in the CLC Genomics Workbench with default parameters and a total minimum count filter cutoff set at 5. An FDR-adjusted *P* value of 0.001 was used as a statistical cutoff to identify the core set of common DEGs. For a detailed analysis of genes involved with central carbon metabolism, the cutoff was increased to 0.05, and those genes with *P* values in the range 0.001 < *P* < 0.05 were marked with an asterisk in Fig. 4c. The map of the *E. piscicida* genome showing the positions of the core DEGs and the T3SS and T6SS loci was generated using circos (67) (version 0.69-5). Heatmaps were generated for genes in the T3SS and T6SS loci using the normalized log2 FPKM + 1 values in the CLC Genomics Workbench. GO term and KEGG pathway enrichment analysis of the core upregulated and downregulated gene sets were done using a hypergeometric test on annotations in the CLC Genomics Workbench. All enriched GO terms in different categories with *P* values less than 0.05 were grouped together, and the top 15 enriched terms (lowest *P* values) in the upregulated and downregulated gene sets were used in generating the plot in Fig. 3a by using the numpy (version 1.24.2), pandas (version 2.0.0), and matplotlib (version 3.7.1) packages in Python (version 3.10.11). The top 10 enriched KEGG pathway annotations were similarly plotted.

For the analysis of antisense RNA expression, trimmed reads were mapped to the EtPo1 genome assembly using bowtie2 (version 2.3.2), and featureCounts (68) (version 1.6.5) was used to generate a count matrix of fragments mapped antisense to coding regions. The count matrix was imported into R (version 4.3.0), and differential expression analysis was done using the edgeR package (version 3.42.2). Detectable antisense transcripts were designated as those that passed a minimum read count cutoff (min. count = 3). An FDR-adjusted *P* value of 0.05 was used as a statistical cutoff to identify the core set of common DEATs. The orientational contexts and intergenic distances of all genes and their upstream and downstream neighbors were determined by an in-house R script that parses the annotated coding strand of the genes from the GFF annotation file of the assembled *E. piscicida* genome. Fold change correlation analyses were conducted in R using the fold changes of the sense and antisense transcripts calculated by the empirical analysis of the DGE method in the different data sets. Scatter plots were generated using ggplot2 (version 3.4.2), and the correlation matrix was generated using corrplot (version 0.92). For predicting excludons by detecting transcript fragments encoding both sense and antisense sequences of genes in convergent and divergent pairs, we extracted read fragments that mapped across the intergenic regions less than 300 bp using pysam (https://github.com/pysam-developers/pysam) in Python. The hypergeometric test for the enrichment of genes in predicted excludons within the set of all DEGs and genes with DEATs was done using pandas, scipy (version 1.11.2), and matplotlib in Python. To generate the stranded RNA mapping visualizations in Integrative Genomics Viewer (IGV) (69) (v. 2.11.9), the strand-specific mapping files generated from the bowtie2 alignment were used to calculate the strand-specific fragments per bins per million mapped reads (BPM) in 10 bp bins using bamCoverage (70) (version 3.5.1) from the deepTools suite. The BPM values were offset by +1, log2 transformed, and averaged across the replicates of each group using WiggleTools (71). The output file was then loaded into IGV for visualization. DEG and DEAT tracks in IGV were loaded from BED files generated by an in-house bash script. GO term enrichment analysis of the genes with upregulated and downregulated antisense transcripts was done using a hypergeometric test on annotations in the CLC Genomics Workbench. All GO terms from different categories with *P* values less than 0.05 were grouped and plotted as above using Python.

### Reverse transcription polymerase chain reaction

To detect overlapping transcription of predicted excludons by RT-PCR, primers were designed within 100 bp upstream of the stop codons or 100 bp downstream of the start codons for genes in convergent or divergent excludons, respectively, using in-house R and Python scripts, and Primer3 (72). Primers to detect 16S rRNA were adopted from previous literature (73). All primers are listed in Table S12. One microgram of RNA extracted from an *E. piscicida* culture grown to an OD_600_ of 1.0 was reverse transcribed into first-strand cDNA using a PrimeScript RT Reagent Kit with gDNA Eraser (Perfect Real Time) (Takara) according to the manufacturer’s instructions with a minor modification, i.e., the DNase reaction was extended for up to 15 minutes. As a negative RT control, 1 µg of RNA was also used in a reaction with no reverse transcriptase enzyme added. One microliter volumes of 10-fold diluted reactions were used for each PCR reaction using a PCR Master Mix 2× (ThermoFisher) according to the manufacturer’s instructions. As positive and negative PCR controls, 1 ng of gDNA and sterile water were loaded as templates, respectively. PCR cycling conditions for all targets were as follows: initial denaturation for 3 minutes at 95°C, followed by 40 cycles of 30 s at 95°C, 30 s at 55°C, and 30 s at 72°C followed by a final extension for 5 minutes at 72°C.

## Data Availability

The sequence data generated in this study have been deposited in the NCBI Sequence Read Archive under NCBI BioProject ID no. PRJNA1001190 with accession numbers SRR25495528–SRR25495539.

## SUPPLEMENTAL MATERIAL

Supplemental Figures mbio.03526-23-s0001.pdf Figures S1 to S11.

Supplemental Tables mbio.03526-23-s0002.xlsx Tables S1 to S12.

ASM does not own the copyrights to Supplemental Material that may be linked to, or accessed through, an article. The authors have granted ASM a non-exclusive, world-wide license to publish the Supplemental Material files. Please contact the corresponding author directly for reuse.
