# Supplementary material for: Transcriptome analysis of Edwardsiella piscicida during intracellular infection reveals excludons are involved with the activation of a mitochondrion-like energy generation program
Source: mBio. 2024 Feb 13;15(3):e03526-23. doi: 10.1128/mbio.03526-23 (PMC10936155; doi:10.1128/mbio.03526-23)
Supplement: Supplemental Figures — Figures S1 to S11. [file mbio.03526-23-s0001.pdf]

Supplementary Figures

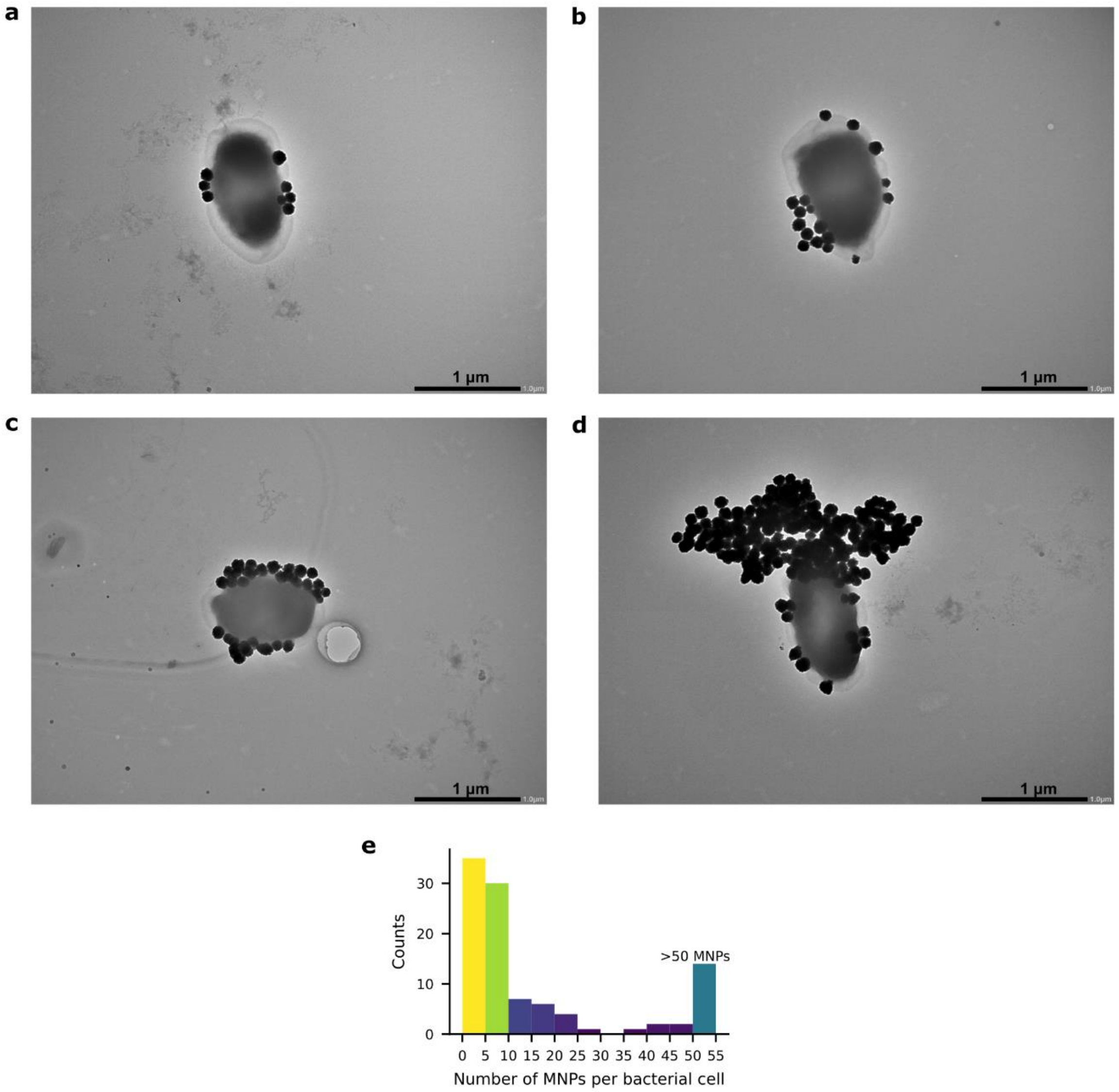

Supplementary Figure 1. Number of MNPs labeled to single bacterial cells. **a-d,** Representative TEM micrographs of single bacterial cells labeled with 8 (**a**), 17 (**b**), 37(**c**), and a large aggregate of greater than 50 MNPs (**d**). **e,** Frequency distribution of the number of MNPs labeled to the surface of bacterial cells. Labeled MNPs exceeding 50 are grouped into one bin. n = 102 bacteria.

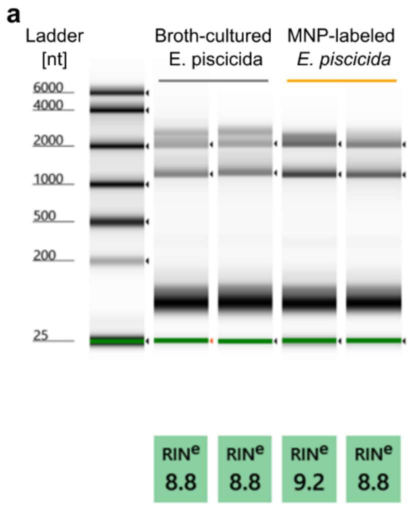

Supplementary Figure 2. Effect of MNP labeling on RNA quality (**a**) Electropherogram of RNA extracted from MNP-labeled bacteria compared to RNA from broth-cultured bacteria. RNA Integrity Numbers (RIN) ranging from 0 (poor quality) to 10 (high quality) are shown below the lanes. Electropherogram lanes are scaled to the highest intensity peak.

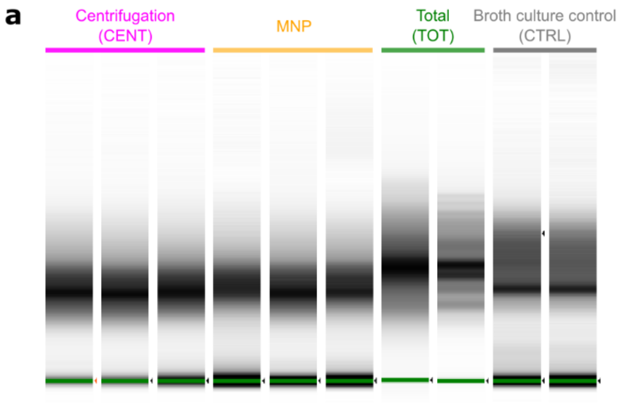

Supplementary Figure 3. a, Electropherogram of rRNA-depleted RNA from *E. piscicida*-infected BRF41 cells extracted by different methods and from broth cultured *E. piscicida*. CENT, centrifugation enrichment method; TOT, total RNA extraction; MNP, MNP enrichment method; CTRL, broth cultured *E. piscicida*.

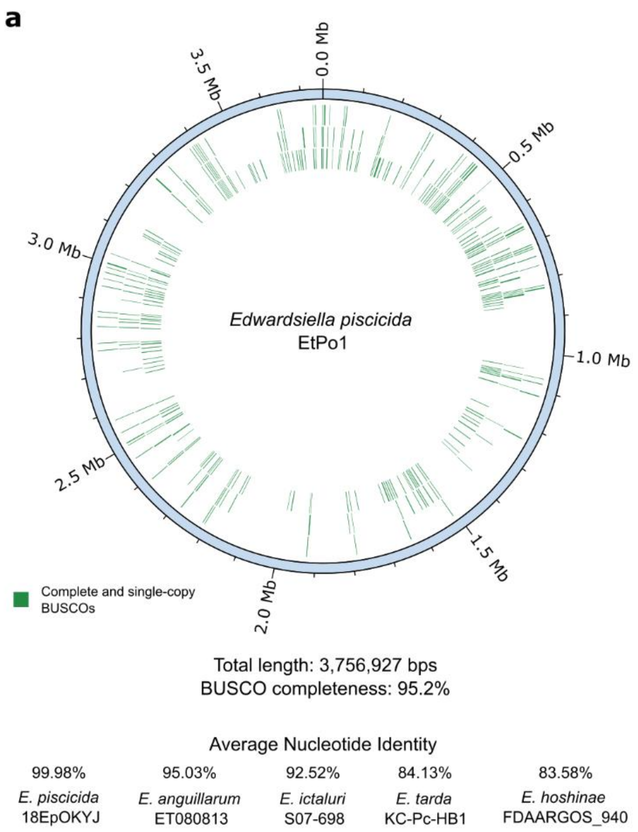

Supplementary Figure 4. The *E. piscicida* EtPo1 genome. **a,** Genome assembly of Oxford Nanopore sequencing data from *E. piscicida* EtPo1. The light blue outer ring represents the bacterial chromosome and the green bars in the inner ring represent the locations of all complete and single-copy BUSCOs. The total chromosome length, BUSCO completeness and average nucleotide identities in comparison to reference genome sequences of *Edwardsiella spp.* are shown at the bottom of the figure.

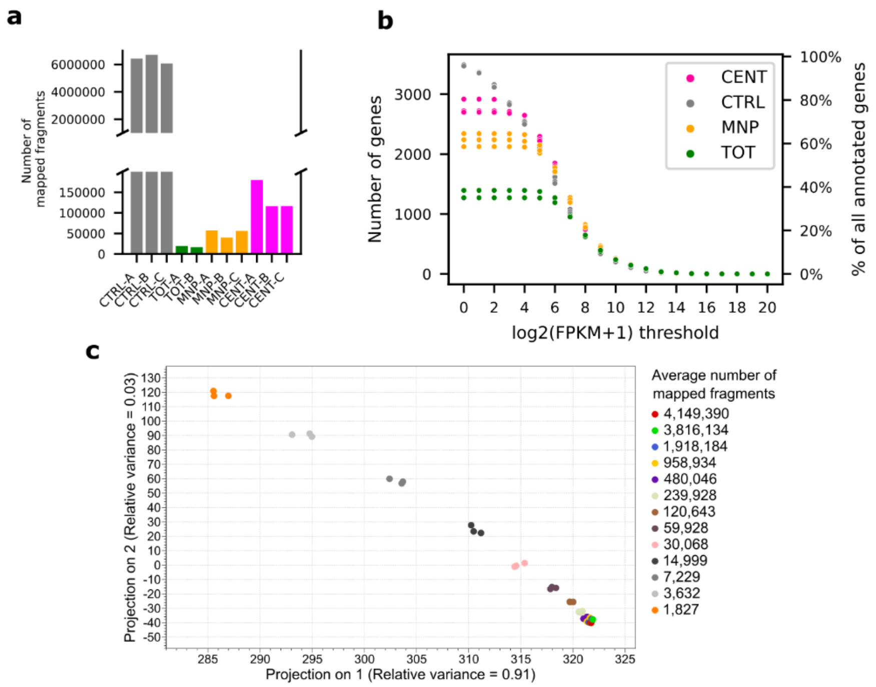

Supplementary Figure 5. a, Total number of fragments mapped to *E. piscicida* genome in enriched (MNP, CENT) and non-enriched (TOT) RNAs from infected BRF41 cells, and from control, broth-cultured bacteria (CTRL). **b,** Number of genes detected at increasing log2(FPKM+1) thresholds from RNA extracted from *E.piscicida*-infected BRF41 cells by different methods and from broth-cultured bacteria. TOT, total RNA extraction; MNP, MNP enrichment method; CENT, centrifugation enrichment method; CTRL, broth-cultured bacteria. **c,** PCA of gene expression data in subsampled reads from sequencing saturated control, broth-cultured *E. piscicida*.

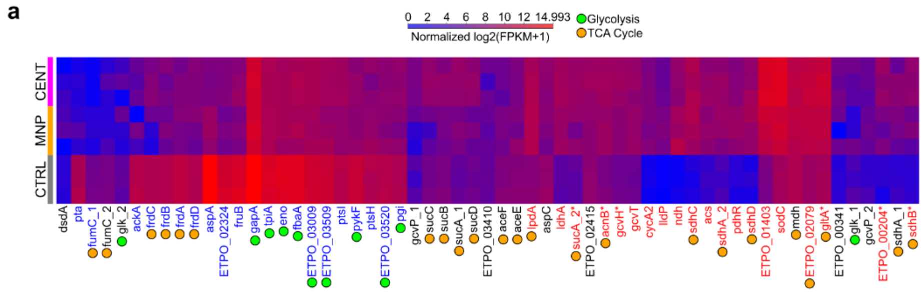

Supplementary Figure 6. a, Heatmap of the normalized fragments per kilobase million (FPKM) expression values of genes involved in glycolysis (green circle), the TCA cycle (orange circle) and central carbon metabolism as in shown in Fig. 4c. Significantly (FDR p-value < 0.001) upregulated and downregulated genes are colored red and blue, respectively. DEGs with near significant (FDR p-value < 0.05) changes in expression levels are marked with an asterisk.

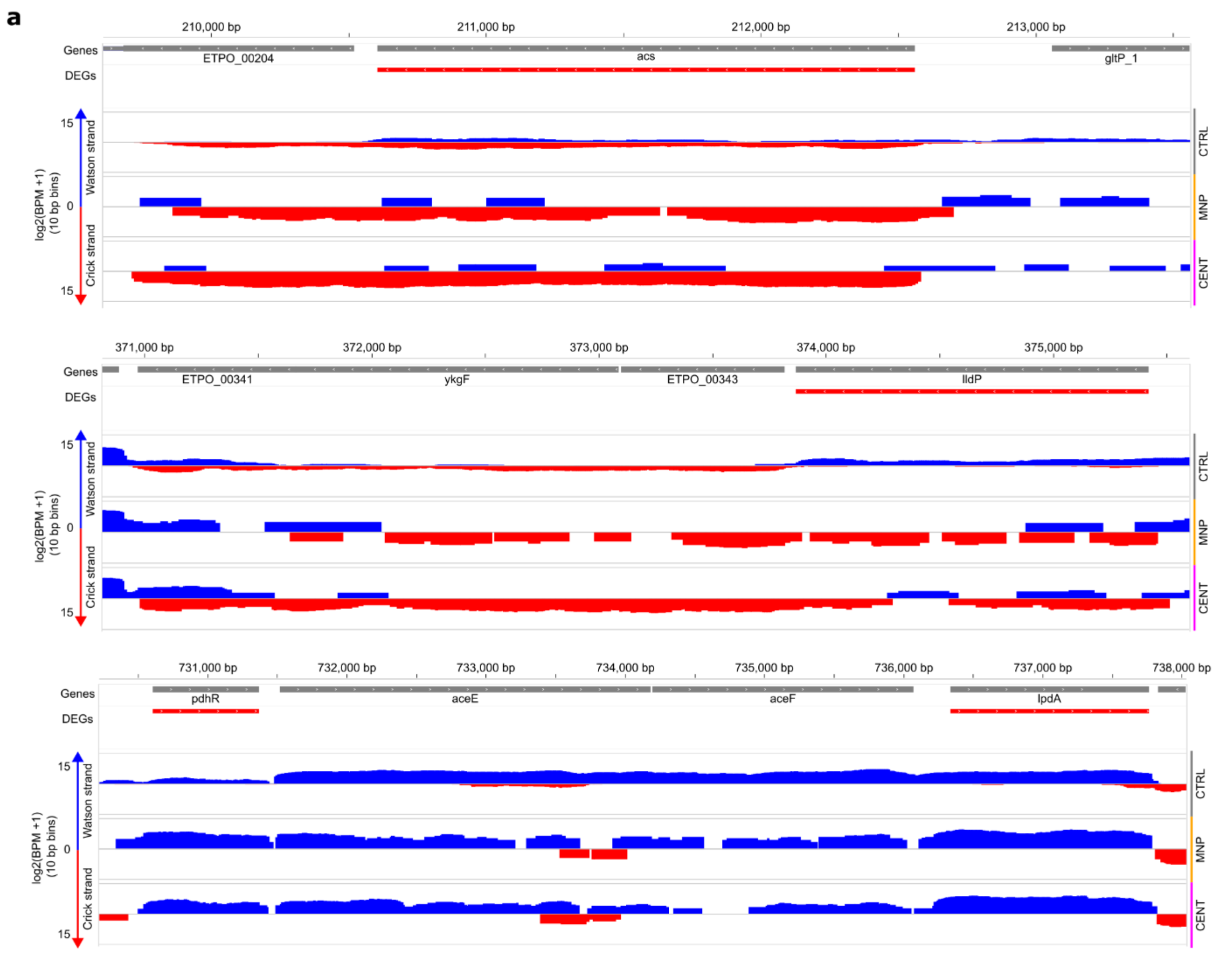

Supplementary Figure 7. Genomic contexts of genes involved with central carbon metabolism in Fig. 4. **a,** The first (upper) panel shows the positions and orientations of genes. The second panel shows DEGs: red, upregulated; blue, downregulated. The last three panels show the mean log2 fragments per bins per million mapped reads (BPM) in 10 bp bins mapped to the Watson (blue) and Crick (red) strands in control bacterial RNAs (grey bar), MNP enriched RNAs (orange bar) and centrifuge enriched RNAs (pink bar).

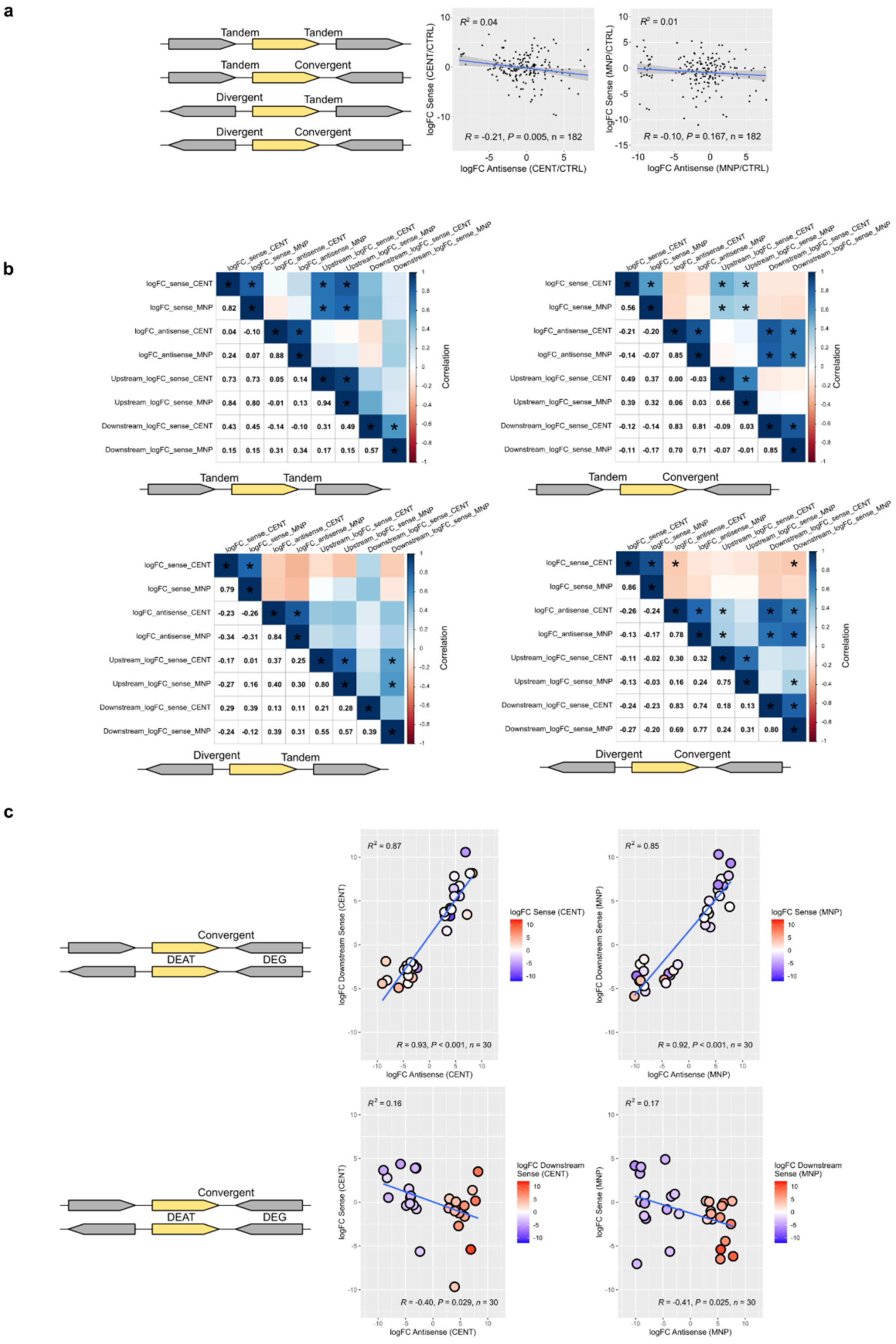

a
Tandem
Tandem
Tandem
Convergent
Divergent
Tandem
Divergent
Convergent
R^2 = 0.04
logFC Sense (CENT/CTRL)
R = -0.21, P = 0.005, n = 182
logFC Antisense (CENT/CTRL)
R^2 = 0.01
logFC Sense (MNP/CTRL)
R = -0.10, P = 0.167, n = 182
logFC Antisense (MNP/CTRL)
b
logFC_sense_CENT
logFC_sense_MNP
logFC_antisense_CENT
logFC_antisense_MNP
Upstream_logFC_sense_CENT
Upstream_logFC_sense_MNP
Downstream_logFC_sense_CENT
Downstream_logFC_sense_MNP
Correlation
Tandem
Tandem
Tandem
Convergent
Divergent
Tandem
Divergent
Convergent
c
Convergent
DEAT
DEG
R^2 = 0.87
logFC Downstream Sense (CENT)
R = 0.93, P < 0.001, n = 30
logFC Antisense (CENT)
logFC Sense (CENT)
R^2 = 0.85
logFC Downstream Sense (MNP)
R = 0.92, P < 0.001, n = 30
logFC Antisense (MNP)
logFC Sense (MNP)
Convergent
DEAT
DEG
R^2 = 0.16
logFC Sense (CENT)
R = -0.40, P = 0.029, n = 30
logFC Antisense (CENT)
logFC Downstream Sense (CENT)
R^2 = 0.17
logFC Sense (MNP)
R = -0.41, P = 0.025, n = 30
logFC Antisense (MNP)
logFC Downstream Sense (MNP)

Supplementary Figure 8. Log-fold change correlation analyses of antisense, sense, upstream counterparts' sense and downstream counterparts's sense transcripts for genes with detectable antisense transcripts. **a,** Genome wide log-fold change correlation analysis for antisense and sense transcripts of genes with detectable antisense transcripts. **b,** For genes in the displayed orientational contexts, correlation matrices of the Pearson's correlations between fold changes of their sense, antisense, downstream counterparts' sense, and upstream counterparts' sense transcripts. Correlations are scaled to color and directly displayed in the upper and lower triangle, respectively. Significant correlations ($P < 0.05$) are marked by an asterisk in the upper triangle. **c,** For responder genes in convergent excludons (see main text), correlation analyses for their antisense and downstream counterparts' sense transcripts (upper), and their antisense and sense transcripts (lower). The dots are colored to the fold changes of their sense transcripts and downstream sense transcripts in the upper and lower plots, respectively.

a

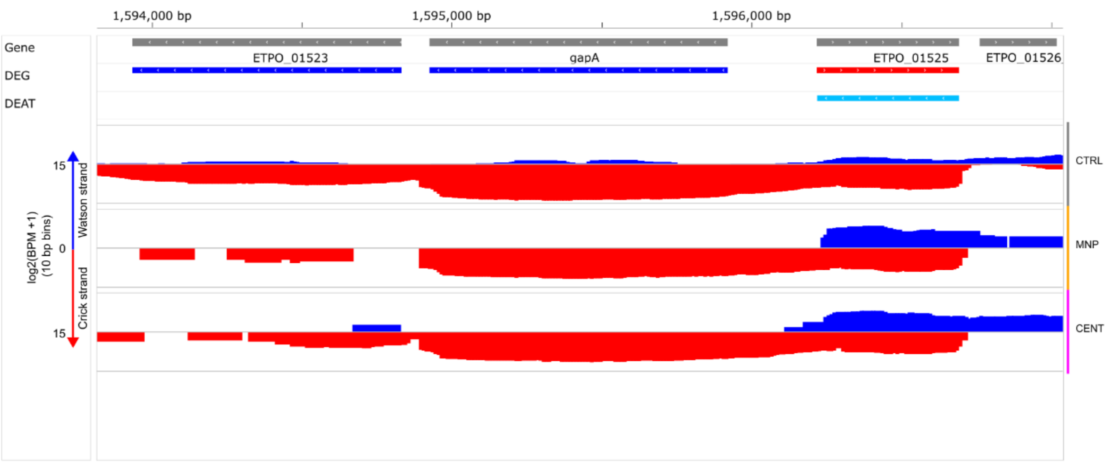

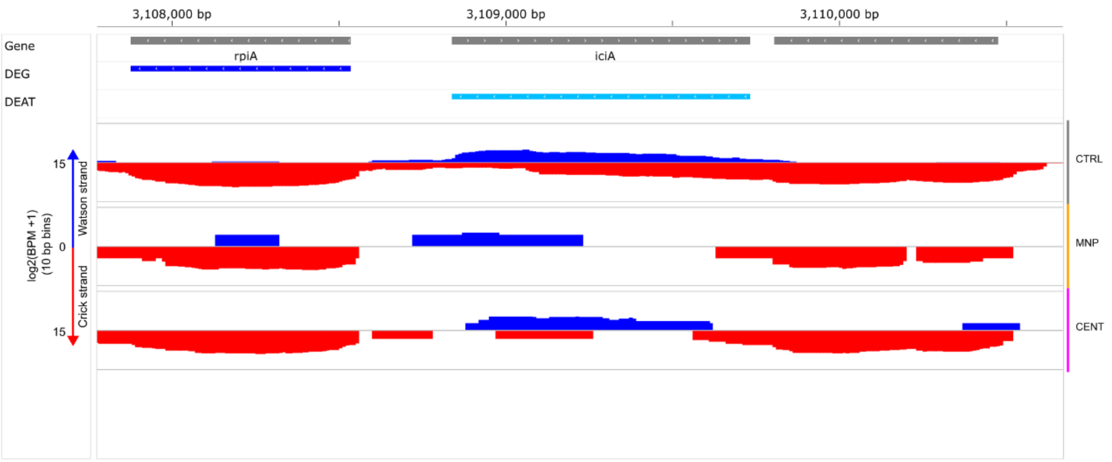

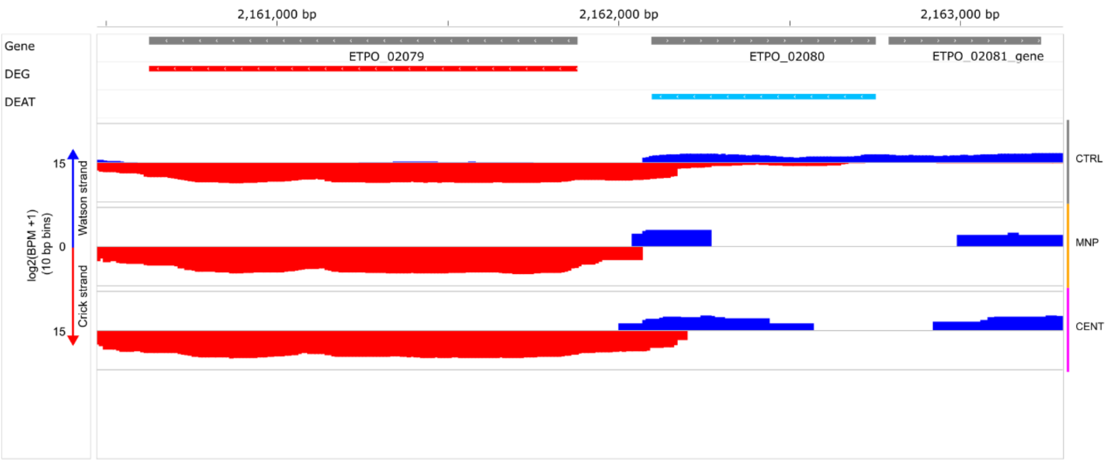

Supplementary Figure 9. Putative responder genes in divergent excludons (ETPO_01525, *iciA*, ETPO_02080). **a,** The first (upper) panel shows the positions and orientations of genes. The second panel shows DEGs: red, upregulated; blue, downregulated. The third panel shows DEATs: light blue, downregulated. The last three panels show the mean log2 fragments per bins

per million mapped reads (BPM) in 10 bp bins mapped to the Watson (blue) and Crick (red) strands in control bacterial RNAs (grey bar), MNP enriched RNAs (orange bar) and centrifuge enriched RNAs (pink bar).

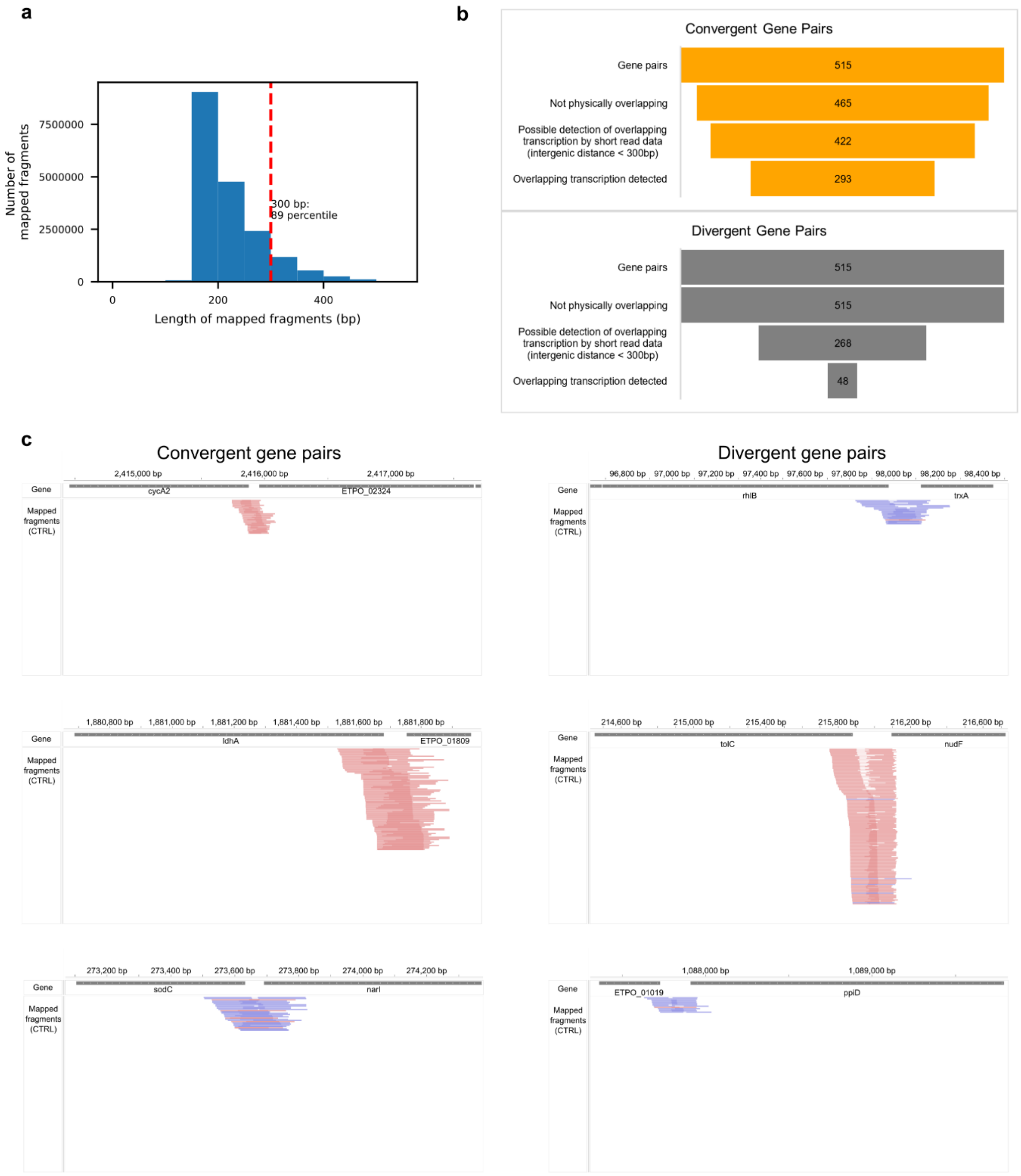

Supplementary Figure 10. Prediction of excludons by detecting transcript fragments encoding both sense and antisense sequences by extracting read fragments that map across intergenic regions. **a,** Histogram showing lengths of mapped fragments across all sequencing libraries from broth-cultured (CTRL) bacteria. The red dotted line shows the percentile at 300 bp. **b,** Funnel chart showing the process and outcomes at each step for predicting convergent (upper) and divergent (lower) excludons. Gene pairs were first filtered for non-overlapping pairs (intergenic distances > 0 bp). These pairs were then filtered to those which overlapping transcription could possibly be detected using short read data (intergenic distances < 300 bp). Positive detection for overlapping transcription was defined as those gene pairs that had at least 1 fragment mapped in

all three CTRL libraries. **c,** Examples of convergent (left) and divergent (right) gene pairs showing overlapping transcript fragments. The upper panel shows the genes and orientations, and the lower panel shows the mapped transcript fragments from all three libraries prepared from the broth-cultured bacteria (CTRL). Fragments mapping to the positive and negative strands are colored blue and red, respectively.

a

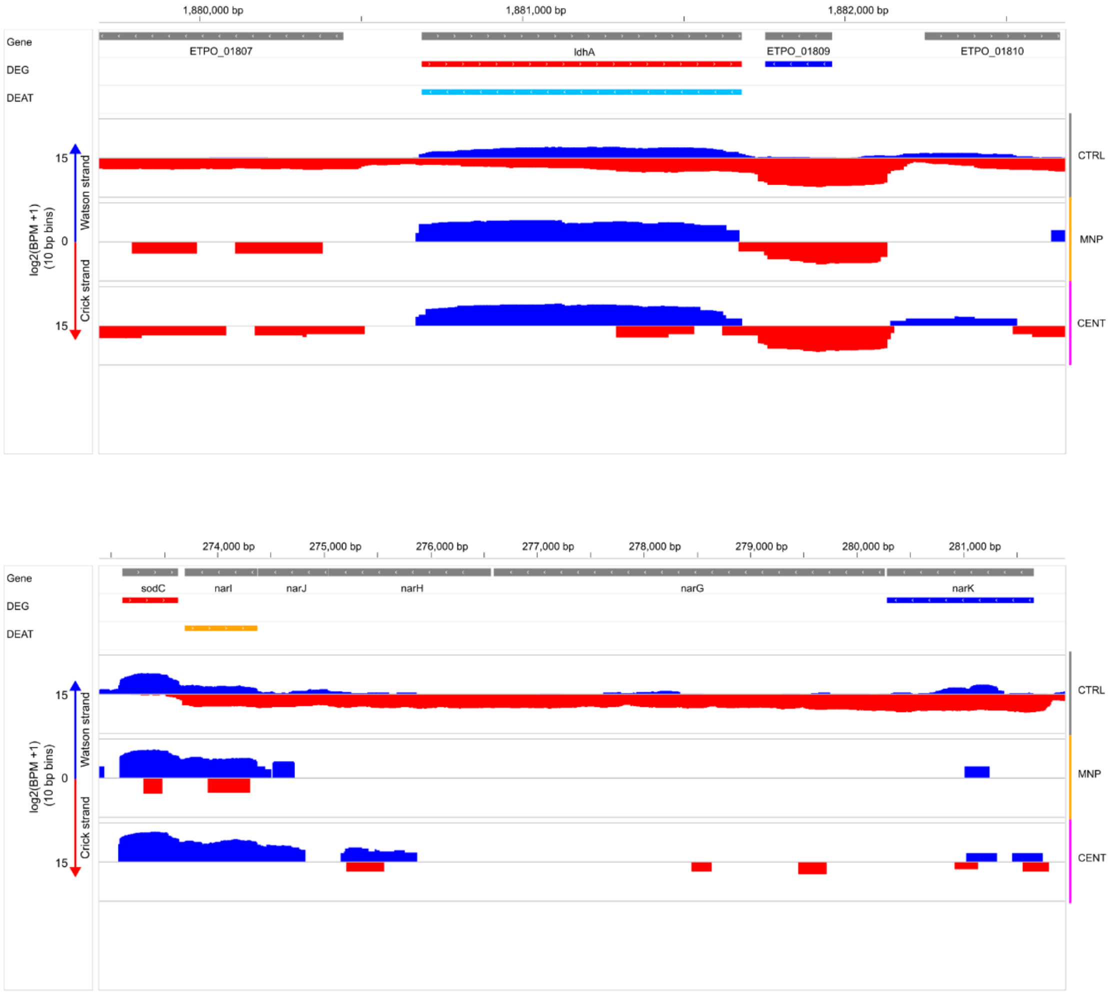

Supplementary Figure 11. Responder genes in convergent excludons (*ldhA* and *sodC*) related to central carbon metabolism. **a,** The first (upper) panel shows the positions and orientations of genes. The second panel shows DEGs: red, upregulated; blue, downregulated. The third panel shows DEATs: light blue, downregulated. The last three panels show the mean log2 fragments per bins per million mapped reads (BPM) in 10 bp bins mapped to the Watson (blue) and Crick (red) strands in control bacterial RNAs (grey bar), MNP enriched RNAs (orange bar) and centrifuge enriched RNAs (pink bar).
